# Molecular and subcellular mechanisms of vital macrophage extracellular trap formation

**DOI:** 10.3389/fimmu.2025.1608428

**Published:** 2025-07-31

**Authors:** Yongchan Lee, Max Brenner, Monowar Aziz, Ping Wang

**Affiliations:** ^1^ Center for Immunology and Inflammation, The Feinstein Institutes for Medical Research, Manhasset, NY, United States; ^2^ Departments of Surgery and Molecular Medicine, Zucker School of Medicine, Manhasset, NY, United States

**Keywords:** macrophage extracellular trap, METs, vital, Gasdermin D, autophagy, LC3, secretory lysosome, ESCRT

## Abstract

Macrophage extracellular traps (METs) are a poorly understood process beneficial for infection control but detrimental in inflammation, autoimmunity and cancer. Our research shows that viable macrophages release METs even when plasma membrane lysis is blocked. We demonstrate, for the first time, that nuclear DNA is extruded directly into the cytoplasm through Gasdermin D pores on the nuclear envelope. Gasdermin D pore formation was triggered by extracellular cold-inducible RNA-binding protein, which activates the TLR4 signal transduction pathway. This DNA is processed in the cytoplasm, enters the vesicular transport system aided by autophagic flux and the Endosomal Sorting Complex. The DNA then enters the lysosomal compartment, where it undergoes histone 3 citrullination, forms nascent traps containing myeloperoxidase, and is released to the extracellular space. Our study provides valuable insights into vital MET formation and its mechanism that will enable future studies on the role of METs in health and disease.

## Introduction

When innate immune cells encounter microorganisms, they respond by releasing soluble mediators such as damage-associated molecular patterns (DAMPs), chemokines, and cytokines, phagocytosing the microbial cells, and producing extracellular traps. Originally described in neutrophils (1), extracellular traps were later identified to be released by other immune cells such as macrophages (2). Macrophages are innate immune cells critical for phagocytosis and antigen-presenting in bacterial infections and cancer. Macrophage extracellular traps (METs) are net-like structures containing chromatin (DNA, histones) along with other proteins (elastase, myeloperoxidase, lactoferrin, etc.) that are released by macrophages when they are exposed to pathogen-associated molecular patterns (PAMPs) on infectious agents or DAMPs from injured cells (3, 4).

We have originally discovered that extracellular cold-inducible RNA-binding protein (eCIRP) released in hemorrhagic shock and sepsis activates immune cells via TRL4 signaling to increase inflammatory responses aggravating organ injury and worsening survival (5). Recently, we showed that eCIRP promotes the formation of Gasdermin D (GSDMD) pores on the plasma membrane of macrophages, leading to pyroptotic METosis (6). The release of pyroptotic METs resembles suicidal NETosis in terms of cell death commitment. In addition to suicidal NETosis, neutrophils can also undergo vital NETosis, triggered by PAMPs and reported in rapid responses to the bacterial infection (7–9), although further investigation is required to fully understand these processes. Vital NETosis involves the vesicular transport of DNA. These processes are distinct from and faster than the suicidal NETosis pathway (10). Furthermore, since vital release of extracellular traps preserves cellular functions such as phagocytosis and cytokine production (10), it is conceivably more inflammatory than suicidal release. It is unclear, however, whether macrophages also undergo vital release of METs, and which molecular and subcellular mechanisms are involved.

In this study, we have showed that macrophages stimulated with eCIRP undergo vital release of METs. We then investigated the formation of vital METs, aiming to thoroughly elucidate the critical molecular mechanisms driving this process. We discovered that nuclear DNA translocate into the cytoplasm of the live cells through GSDMD pores formed on the nuclear envelope. The nuclear DNA is then sequestered into the vesicular system and subsequently transported through the autophagic pathway until it is finally processed and released to the extracellular space through the secretory lysosomal pathway. Identification of mechanisms of METs release in live macrophages provides a solid foundation for future studies on their role in the pathophysiology of in infectious diseases, inflammatory and autoimmune disorders, cancer, and cell-to-cell communication.

## Materials and methods

### Reagents

Recombinant murine CIRP (eCIRP) was prepared in-house as described previously (5). Hoechst 33342 (cat. no. R37605), SYTOX Orange (cat. no. S11368), antibodies against AIM-2 (cat. no. MA5-38442), Rab8a (cat. no. 50-191-613), and TSG101 (cat. no. PA5-31260) were purchased from Thermofisher Scientific (Waltham, MA). Antibodies against cleaved Gasdermin D (cat. no. 36425), CD63 (cat. no. 52090), cGAS (cat. no. 79978S), Lamin A/C (cat. no. 4777S), LAMP-1 (cat. no. 15665S), LC3B (cat. no. 3868S) was purchased from Cell Signaling Technology (Danvers, MA), anti-MPO-FITC (cat. no. ab90812), and anti-histone H3 (citrulline R2 + R8 + R17) antibody (cat. no. ab5103) were purchased from Abcam (Waltham, MA). Antibodies against CHMP4B (cat. no. 13683-1-AP) and CHMP7 (cat. no. 16424-1-Ap) were purchased from Proteintech (Rosement, IL). Glycine and chloroquine were purchased from Sigma-Aldrich.

### Cell culture

Human monocytic cell line THP-1 cells were obtained from ATCC (TIB-202) and maintained in RPMI media with fetal bovine serum (10%), penicillin–streptomycin, and 2-mercaptoethanol (50 µM). The cells were differentiated with 90 ng/ml phorbol 12-myristate-13-acetate (PMA) for 24 h, followed by resting for 24 h in regular media without PMA. The cells were then replenished with fresh media immediately before any treatment was used in this study. THP1-Difluo™ hLC3 reporter cells are purchased from Invivogen. These cells, derived from the THP-1 human monocytic cell line, express the RFP::GFP::LC3 fusion protein in which the human LC3B (microtubule-associated protein 1 light chain 3 beta) is fused to two fluorescent reporter proteins: RFP (acid-stable) and GFP (acid-sensitive). Cell culture samples were collected at 8 and 16 h after treatment of eCIRP unless otherwise indicated. The final concentration of eCIRP was 1 μg/mL. For THP-1 cells transduced with RFP::GFP::LC3 fusion protein the final concentration of eCIRP was 2 μg/mL. Glycine and chloroquine were treated for 30 min prior to the treatment of eCIRP. The incubation of all staining dyes was done 30 min before the treatment of eCIRP. The final concentration of glycine and chloroquine were 25 mM and 20 μM.

### Time-lapse microscopy

Time-lapse microscopy was performed with a Nikon Eclipse Ti microscope equipped with a motorized stage, a DS-Qi2 monochrome camera, perfect focus system, and SOLA 6-LCR-SC lightbox (Lumencor light engine). For live-cell imaging, an EVOS onstage incubator system (Thermofisher Scientific) was mounted on the motorized stage for maintaining temperature (37°C), humidity (80% or higher), and gas supply (5% CO_2_). Nikon Element advance research software was used for image acquisition and quantitative analysis. Time-lapse images were recorded for 16 h at the interval of 15 min. DAPI, FITC, Texas Red, and Cy5 filters were used for detecting different fluorophores used in this study. Differential Interference Contrast (DIC) imaging is always acquired together with fluorescence imaging. Hoechst33342 staining was detected only one time by UV light illumination and DAPI filter together with DIC image at the beginning of the time-lapse imaging. The nuclear images obtained with Hoechst33342 staining were used for counting initial cell numbers, which is used to normalize the result of other fluorescence imaging in each microscopic field. Quantitative analysis was done by Nikon Element analysis, including thresholding, binary layer, and automated measurement module. For live-cell imaging, 35 mm glass-bottom culture dish (part no.: P35G-1.5-20-C), 6-well (part no.: P06G-1.5-20-F, and 12-well (part no.: P12G-1.5-14-F) glass-bottom culture plates were used (MatTek Life Sciences).

### Immunofluorescence

Cells were fixed with 4% paraformaldehyde for 15 min at room temperature and washed 3 times with PBS. The fixed cells were then blocked with 5% BSA and 100 mM glycine in PBS for 2 h, followed by the incubation of the primary antibody overnight at 4°C. Antibody was mixed in the blocking buffer. The cells were then washed 3 times with PBS and incubated with fluorescence tagged secondary antibody for 2 h. After washing the cells 3 times with PBS, the immunofluorescence specimen was prepared by adding prolong gold antifade (cat. no.: P36934, Thermofisher Scientific).

### Confocal microscopy

High-resolution images were obtained by a Axio Observer.Z1/7 equipped with Zeiss LSM900 confocal microscopy system. The z-stack images of cells were acquired with Plan-Apochromat 63×/1.40 Oil DIC M27 objective lens. SR-4Y fast acquisition mode of Airyscan and 4× averaging was used. The images obtained by confocal microscope were merged and combined by FIJI ImageJ (11). 3-D volume view and movie were generated by Imaris software (Oxford Instruments).

### Quantitative image analysis

Confocal images with immunofluorescence staining were analyzed with FIJI ImageJ. Low magnification with Plan-Apochromat 10X/0.45 M27 objective was used for the imaging. The stitched images of 9 field of view covers area of about 2000 cells in each sample. Nuclear counting was done with Hoechst33342 images. Citrullinated Histone3 (CitH3) quantification was done with immunofluorescence staining with anti-citrullinated Histone3 antibody. Briefly, nuclear condensation counting was done recording ROIs by particle counting command using binary segmentation generated with multiple processes including threshold, close, fill hole, and watershed. The meta data of the ROI measurement was obtained including nuclear area, and mean fluorescence intensity. The data file was exported as a csv data file. The dot plot of MFI vs. nuclear area was generated by FlowJo software with FCS files converted from the csv data. The condensed nuclei were identified as we gated the nuclear objects with high in MFI of Hoechst33342 and smaller in the 2-dimensional area of the nuclei in the field. To measure CitH3 signal only in the METs area the CitH3 signal in the nuclear region was removed by applying ROI of nuclear region and fill the ROI with black color. Then, the segmentation of METs region was done by generating a binary mask with thresholding command and the resulting segment of METs region was counted by particle analysis command. The resulting segmentation frequently showed multiple METs particles around a cell. To minimize overestimation of the proportion of METs over total cells due to the multiple segments around a single cell, the binary mask was dilated three times to merge segments in close proximity. The percentage and fold ratio of resulting METs count was calculated by the number of nuclei in the field, which is used for counting total number of cells. For the quantification of DNA puncta positive cell number over the total cells in the microscopic field we acquired high-resolution images using 63× objective and stitched 20 consecutive images. The cell boundary was identified with MPO antibody staining and the whole cell boundary in the image was recorded as a ROI set. Using this ROI set the DNA puncta in each cell were detected and quantified with SYTOX Orange staining. The nuclear staining by hoechst33342 was used to exclude the SYTOX Orange staining of the nucleus in the image field. This was done by masking the SYTOX Orange staining image with the binary ROI overlay of the nuclear staining. We then applied ROI set of cell boundary to the binary image of SYTOX Orange staining and counted the DNA puncta using particle analyzer of ImageJ.

### Colocalization analysis

Confocal images with immunofluorescence staining were analyzed with FIJI ImageJ. High resolution single cell image by z-stack were acquired with Plan-Apochromat 63×/1.40 Oil DIC M27 objective lens. SR-4Y fast acquisition mode of Airyscan and 4× averaging was used. Single slice of z-stack was selected for colocalization. For colocalization analysis, images of 3 to 8 cells from each treatment group were used. The ImageJ plugin, colocalization highlighter, was used for generating a representative picture of colocalization point, and JACoP plugin was used for the calculation of Manders’ coefficient (12). The fluorescence signal of cellular proteins is tested with DNA signal in the cytoplasm. To measure the colocalization of cytoplasmic DNA and protein signal in the whole cell images the nuclear signal of DNA and protein staining was removed from the images. Then, the threshold of both images was adjusted appropriately. For Manders’coefficient, we summarized only the portion of the cytoplasmic DNA colocalized with the protein fluorescence signal subjected to the analysis. It is due to the absence of cytoplasmic DNA in the control cells, and we couldn’t calculate the portion of cellular protein signal colocalized with the DNA, which does not exist in the cytoplasm of the control cells.

### Western blotting

The lysate of THP-1 cells was prepared by adding RIPA buffer to each sample. The RIPA buffer was supplemented with 2 mM Sodium Orthovanadate, 0.2 mM phenylmethylsulfonyl fluoride (PMSF), and cOmplet mini-protease inhibitor cocktail from Roche (cat. No.: 11836153001; Millipore, Sigma, St. Louis, MO). The protein concentration of the lysate was determined with a protein assay reagent (cat. no.: 5000002, Bio-Rad, Hercules, CA). Protein samples were separated by SDS-PAGE using 4–12% Bis-Tris gel (Invitrogen, NP0322, Thermofisher Scientific) and transferred to nitrocellulose membrane by X cell II blot module. The membrane was incubated with primary antibody overnight at 4°C, followed by the incubation of Odyssey secondary antibody (cat. no.: 926–32211 or 926-68070, Lycor Biosciences, Lincoln, Nebraska). The detection and quantification of Western blot was done by Odyssey CLx imaging system (Lycor Biosciences).

### Nuclear extract preparation

Five million human macrophage THP-1 cells were seeded in 10 cm culture dish. The culture plate was placed on an ice bath for 10 min prior to the extraction process. The cells were washed three times with ice cold PBS and nuclear extraction was performed according to the procedure described in the nuclear extraction kit from Abcam (cat. No.: ab113474). Briefly, the cells were incubated with pre-extraction buffer and scraped in the buffer and subjected centrifuge, 200xg for 10 min at 4°C. The cells were resuspended with pre-extraction buffer and vortexed briefly and incubated in ice for 20 min. The sample was centrifuged with 750xg for 10 min and the pellet was collected. The pellet was resuspended in nuclear extraction buffer and sonicated briefly.

### Statistical analysis

Data represented in the figures are expressed as mean ± SEM. The two-tailed Student t-test was used for two-group comparisons and one-way ANOVA with *post-hoc* Tukey test was used for multiple-group comparisons. A statistical significance threshold was considered for p ≤0.05 between study groups. Normal distribution of the experimental dataset was tested with quantile-quantile plot analysis (**Supplementary Figure S11**). Data analyses and the graph preparation were carried out using GraphPad Prism graphing and statistical software (GraphPad Software, San Diego, CA).

## Results

### Viable macrophages release extracellular traps DNA after stimulation with eCIRP

**Figure 1:**
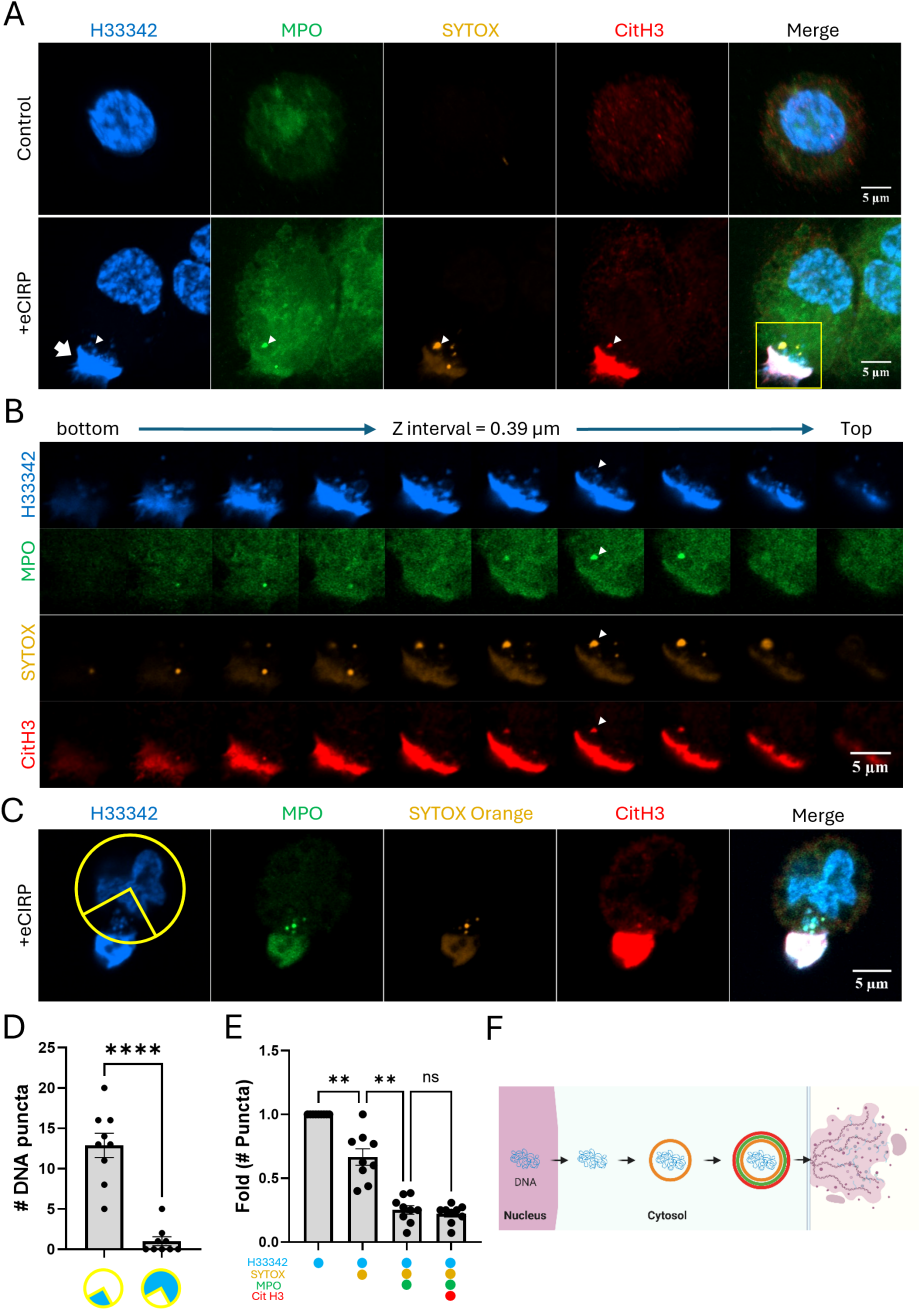
METs are released from live macrophages. The release of METs from live THP-1 macrophages stimulated with eCIRP(1μg/mL) were observed by confocal microscopy, **(A)** Confocal z-stack images were processed by maximum intensity projection. The DNA was stained with Hoechst33342 and SYTOX Orange in the cell culture media. The immunostaining for MPO and citrullinated Histone3 was done after the fixation of the cells. The puncta in the cytoplasm were differentially stained with markers and it was distinguished by the z-stack series of the images, **(B)** The METs were stained intensely with all 4-color staining, arrow. Among the DNA puncta in the cytoplasm, a punctum was positive to all 4-color staining was observed, arrowhead. The cells were analyzed for their polarized distribution of nuclear DNA puncta in their cytoplasm, **(C)** Based on the position of the Extracellular DNA deposited on the edge of the cells (n = 9/group), a quadrant of a circular ROI was superimposed in between the METs and the center of the nucleus, and each nuclear DNA puncta throughout the z-stack images were counted and summarized in the graph, **(D)** The scale bars in the images are 5 μm in size. Student *t*-test: ****p < 0.0001 vs. 1 quadrant where the METs is located. From the same set of cell data, the differential staining of the puncta was summarized the number of puncta with each staining combination were normalized to the ratio of total number of puncta observed from the cells, **(E)** One-way ANOVA: ns, not significant, ** < 0.001. The stepwise processing events for DNA puncta in the cytoplasm were depicted as a schematic diagram, **(F)** Each color circle around the DNA depicts the dye staining corresponding to the immunostaining observed in the confocal image.

Macrophages were stimulated with eCIRP and stained SYTOX Orange prior to cell fixation. SYTOX Orange is a DNA-binding dye that selectively stains dead cells with compromised plasma membrane/nuclear envelope integrity. Macrophages stimulated with eCIRP released nuclear DNA to the extracellular space. The extracellular DNA had strong SYTOX Orange staining, which was done in the culture media prior to the fixation of the cell. The cell nucleus, however, was not significantly stained (**Figure 1A**), indicating that the cell maintained its plasma membrane integrity and remained viable. Interestingly, nuclear DNA released in the cytoplasm of the cell showed selective staining to SYTOX Orange (**Figure 1A**, bottom panel). The z stack series of the same cell showed the spatial distribution of the METs and DNA puncta in the cell (**Figure 1B**). The DNA puncta were situated in the cytoplasmic space between the nucleus and the site of extracellular traps released (**Figure 1C**). The distribution was analyzed with 9 z-stack confocal images, and they showed highly polarized distribution of the DNA puncta toward where the bulk of extracellular traps DNA is observed (**Figure 1D**, **Supplementary Figure S1**). Further analysis of each DNA punctum in the cytoplasm showed stepwise staining of DNA puncta: first only Hoechst33342, then also SYTOX Orange, then additionally by MPO and CitH3 (**Figure 1E**). Taken together, these observations suggest an active process of directional trafficking of the DNA puncta from the nucleus and through the cytoplasm, likely involving the vesicular transport system, and then to the extracellular space (**Figure 1F**).

### eCIRP induces macrophage pyroptosis

**Figure 2:**
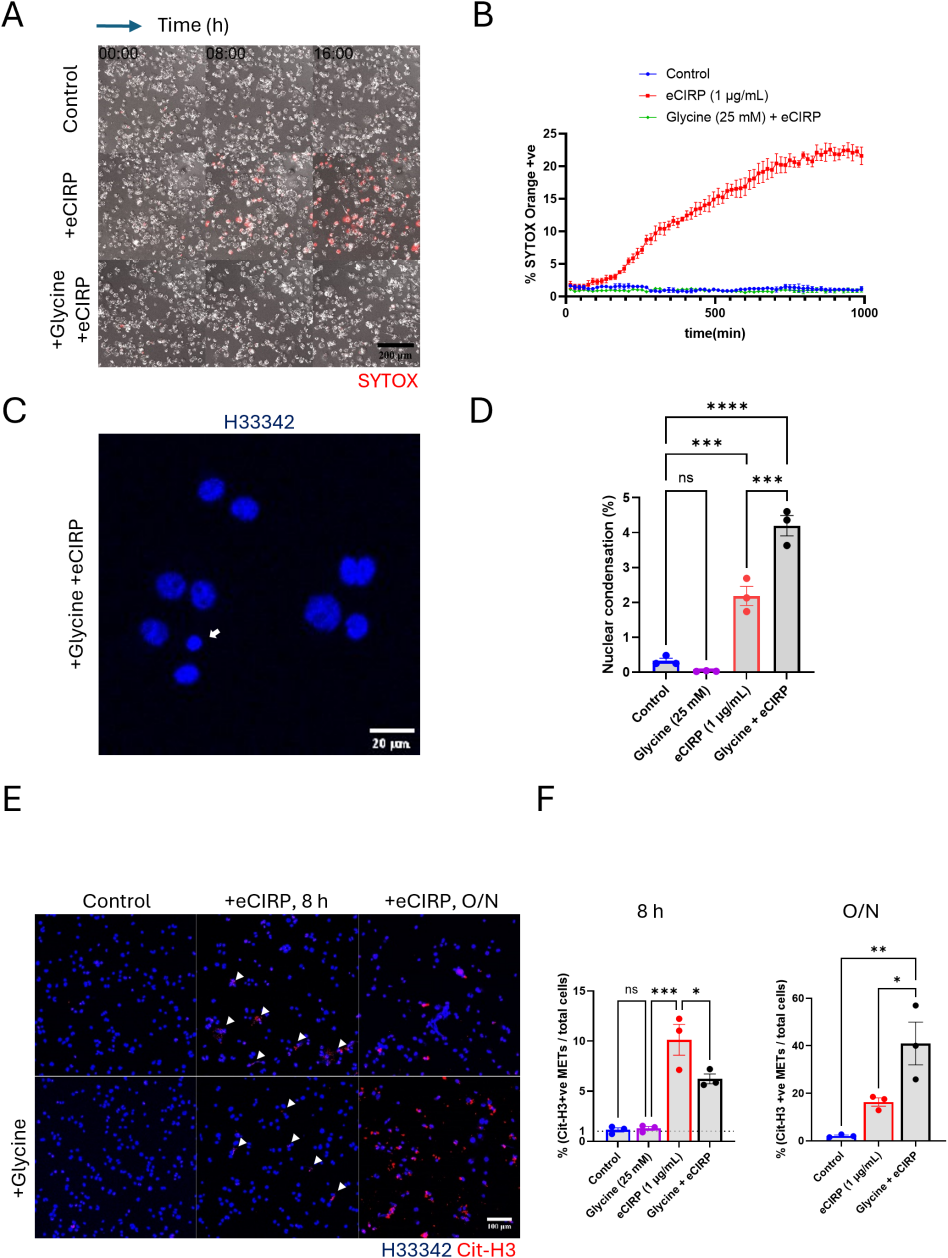
METs release from live cells was independent from the plasma membrane rupture. The pyroptotic cell death by eCIRP (1μg/mL) was assessed by live cell time-lapse imaging, **(A)** The live imaging was recorded every 15 min time interval for 16 h after the treatment of eCIRP. Glycine treatment was done about 30 min prior to eCIRP treat. The cell death was measured by the increase of SYTOX Orange fluorescence in the field of microscope. The scale bar in the image is 200 μm in size. The cells positive to SYTOX Orange dye were automatically counted by the Element software for Nikon microscope and normalized with the initial number of cells, which is counted by the nuclear DNA staining with Hoechst33342, **(B)** The cells treated with glycine (final concentration 25 mM) were not stained by SYTOX Orange and had identical curve to that of Control experiment. Each data point in the live imaging data is the average of 3 different field in the same sample well. Nuclear condensation was measured by confocal microscopy of fixed cells, **(C)** The DNA staining dye, Hoechst33342 was used to stain the macrophages and the confocal images with 10X objective were taken for the analysis of nuclear shape of the macrophages. Scale bar is in size of 20 μm. The small and densely stained nuclei were quantified, and the data was analyzed by FlowJo software, **(D)** One-way ANOVA: ns, not significant, *** < 0.001, and **** < 0.0001. Immunofluorescence images for citrullinated Histone3 staining was used for the measure of METs release from the macrophages. The data was assessed at two different length of incubation time: 8 h incubation and 16 h, overnight (O/N), incubation, **(E)** Scale bar is 100 μm in its size. The percentage of the METs number over total cell number was assessed and compared to the spontaneous METs release of the control cells, **(F)** One-way ANOVA: ns, not significant, * < 0.05, ** < 0.01, and *** < 0.001.

Exposure to eCIRP binds to TLR4, activating downstream signaling pathways and the inflammasome, which can lead to macrophage pyroptotic death (6). This plasma membrane rupture can be easily detected by SYTOX Orange dye. Indeed, our live imaging time-lapse showed that about 20% of the macrophages stimulated with eCIRP underwent pyroptotic cell death (**Figures 2A, B**, **Supplementary Movie S1**). The number of SYTOX Orange positive cells reached a plateau at around 10 h after eCIRP treatment. These pyroptotic METs were easily detectable in a low-power field of the live imaging because once the plasma membrane ruptures the dead cells release a large amount of DNA easily detectable as METs by SYTOX Orange staining.

To distinguish the vital release of METs from suicidal MET formation, we treated the cells with glycine (final concentration: 25 mM). Glycine is an inhibitor of Ninjurin-1 (NINJ1) clustering, which mediates plasma membrane rupture (cytolysis) in programmed cell death, such as pyroptosis and necroptosis (13–17). Addition of glycine to the macrophages abolished the pyroptotic cell rupture induced by eCIRP (**Figures 2A, B**), significantly reducing the number of SYTOX Orange positive cells to the same level of unstimulated control macrophages. Beside the pyroptosis, from the nuclear images of cells treated with glycine and eCIRP together stained with Hoechst33342, we analyzed the nuclear size and its fluorescence intensity to ascertain nuclear condensation, which indicates apoptotic cell death. Macrophages treated with eCIRP alone showed 2% of cells underwent apoptosis. Glycine cotreatment with eCIRP showed only 2% increase in nuclear condensation compared to the cells eCIRP alone treated (**Figures 2C, D**, **Supplementary Figure S2**). These results indicate that eCIRP-mediated cell death is predominantly pyroptosis and could be suppressed effectively.

### DNA release from viable cells is independent of plasma membrane rupture.

Our live imaging time-lapse showed that 80% of the macrophages stimulated with eCIRP did not show cell death, which was detected by SYTOX Orange staining. The vital release of METs generated by these viable cells could only be observed by a high-power field under the confocal microscope due to their small size of DNA. To further characterize the DNA release from viable cells, the METs were quantified using the fluorescence image of anti-CitH3 antibody staining. Both 8 h and overnight treatment samples showed CitH3 positive extracellular traps released by stimulation with eCIRP (**Figure 2E**). After 8 h of stimulation with eCIRP, glycine reduced MET formation to approximately half that of macrophages stimulated with eCIRP alone. Remarkably, METs were still released without plasma membrane rupture (**Figures 2E, F**). The increase in cells releasing METs with eCIRP was only 6% (from 10% at 8 h to 16% after overnight incubation) whereas glycine treatment increased it significantly from 6% to 41%. The difference between eCIRP group and cotreatment group of glycine and eCIRP was 25%. After overnight incubation, cells cotreated with glycine and eCIRP showed 3 times more METs than that of cells treated with eCIRP alone, whereas cells with eCIRP alone didn’t show any more increase in METs release after 8 h. This indicates that non-lytic, viable cells contributed to the increased release of METs.

### Nuclear DNA release is mediated by activated Gasdermin D on the surface of the nuclear envelope.

**Figure 3:**
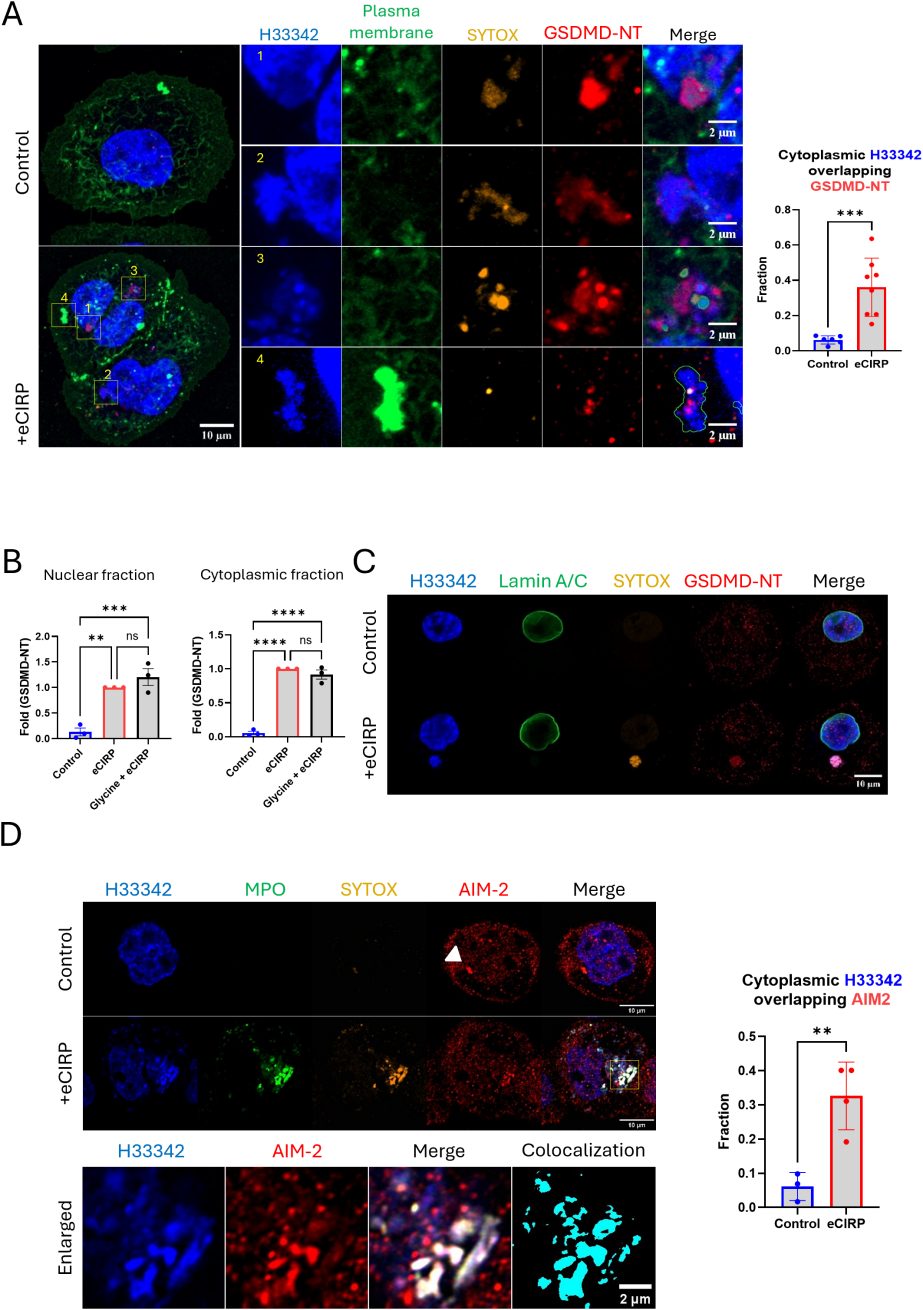
Nuclear DNA release from the nucleus to the cytoplasm through GSDMD pores. Nuclear targeting of the cleaved Gasdermin D was assessed by the immunofluorescence assay against anti-GSDMD-NT antibody, which specifically binds to Asp275 residue of GSDMD-NT, **(A)** For colocalization analysis of control cells (n = 6) and eCIRP-treated cells (n = 8), Manders’ coefficient was evaluated and showed significant colocalization of cytoplasmic DNA and GSDMD-NT by eCIRP treatment. Student *t*-test: ***p < 0.001. The treatment of eCIRP to the macrophage showed increased staining of anti-GSDMD-NT antibody. The image has two cells attached each other. There are 4 enlarged images organized in the figure. Those enlarged images showed nuclear DNA in morphologically and spatially distinct. 1: The nascent DNA extrusion was caught in the image. 2: the DNA was clearly shown released but still connected to the nucleus. 3: the DNA was separated completely from nucleus and densely pack in the form of puncta. 4: DNA released out of the cell was shown co-localized with the plasma membrane staining, which labels the proteins on the surface of the cell. Due to visualizing the membrane ruffle on the cells the membrane staining signal on the region where extracellular DNA was released was extremely high. The plasma membrane staining in the merge picture was only outlined in the image 4 so that the extracellular DNA and other fluorescence signals was visualized at the same time. Scale bar for the whole cell picture and enlarged pictures are 10 μm and 2 μm respectively. For colocalization analysis of control cells (n = 6) and eCIRP-treated cells (n = 8), Manders’ coefficient was evaluated and showed significant colocalization of cytoplasmic DNA and GSDMD-NT by eCIRP treatment. Student *t*-test: ***p < 0.001. The targeting of GSDMD-NT in the nucleus was confirmed by the western blotting with nuclear fraction prepared from the macrophages treated with eCIRP, **(B)** The data was the result of 3 biologically independent experiments. One-way ANOVA: ns, not significant, ** < 0.01, *** < 0.001, and **** < 0.0001. Lamin A/C was not detected in the DNA mass released to the cytoplasm. Lamin A/C was only present in the edge of nucleus, **(C)** Only the released DNA was stained with the SYTOX Orange dye and positive to the anti-GSDMD-NT antibody. Scale bar is 10 μm. Further immunofluorescence assay was done with anti-AIM-2 antibody staining. The AIM2 speckle in the control image was clearly visualized. No DNA in the cytoplasm was observed in the control cell. DNA released was positively stained with AIM-2 after the treatment of eCIRP. the enlarged image showed significant colocalization between DNA and AIM2 in the cytoplasm, **(D)** Scale bar is 10 μm. Unlike control cells, the AIM2 speckle was not observable clearly in the cell treated with eCIRP. For colocalization analysis of control cells (n = 3) and eCIRP-treated cells (n = 4), Manders’ coefficient was evaluated and showed significant colocalization of cytoplasmic DNA and AIM2 by eCIRP treatment. Student *t*-test: **p < 0.01.

We performed immunofluorescence of GSDMD-NT in the live cells and observed the colocalization of nuclear DNA released and GSDMD-NT protein (**Figure 3A** and **Supplementary Movie S2**). We used GSDMD-NT specific antibody (Asp275) in this experiment. GSDMD-NT was detected in multiple places in the cells, including the cytoplasm, nucleus, and plasma membrane. The blown-up images revealed different stages of DNA release, based on the position of each nuclear DNA punctum, and clearly showed that they were accompanied by GSDMD. Image 1 shows GSDMD-NT concentrated in the immediate proximity of the nucleus and co-stained with SYTOX. The 3-D reconstruction movie clearly showed that GSDMD-NT encircles the DNA being extruded from the nucleus. It was observed there was significant gap of Lamin A/C (nuclear membrane) staining at the site where the DNA extrusion was observed (**Supplementary Figure S3**). The protrusion of nuclear DNA was further elongated in image 2 (**Figure 3A**, **Supplementary Figure S3**). A notable observation is that the circular DNA puncta in image 3 showed the accumulation of SYTOX Orange and the antibody staining. The plasma membrane staining in this experiment was done by labeling the cell surface protein with green fluorescence dye. The DNA released from nucleus in image 4 of **Figure 3A** was densely packed with plasma membrane staining dye, which indicates the DNA has been exposed to the extracellular space. Colocalization analysis showed a significant increase in the fraction of cytoplasmic DNA colocalized with GSDMD-NT. Control cells showed average colocalization fraction of 0.06, but in the treatment group it was 0.36. To further verify the nuclear targeting of GSDMD-NT we quantified the GSDMD-NT by Western blot of eCIRP treated macrophages in three biologically independent experiments and showed that GSDMD-NT protein was detected in both nuclear and cytoplasmic fractions of eCIRP treated macrophages (**Figure 3B**, **Supplementary Figure S4**). As expected (14), glycine did not alter the activation of GSDMD.

### Nuclear DNA is released straight into the cytoplasm without any membrane coating.

Based on the colocalization of GSDMD-NT and the released nuclear DNA, we hypothesized that the DNA passed from the nucleus directly into the cytoplasm through GSDMD pores. To evaluate whether the nuclear DNA is released to the cytoplasm encased in vesicles containing remnants of the nuclear envelope, we assessed the puncta for the presence of anti-Lamin A/C antibody and showed that the released DNA with GSDMD does not containing Lamin A/C (**Figure 3C**). We next assessed whether this DNA could be detected by a cytoplasmic DNA sensing protein and showed that the DNA punctum co-stained with GSDMD-NT and SYTOX Orange. We also evaluated the ability of AIM-2 to detect bare nuclear DNA in the cytoplasm using an immunofluorescence assay. Indeed, AIM-2 significantly colocalized with the DNA in the cytoplasm (**Figure 3D**). Colocalization analysis showed a significant increase in the fraction of cytoplasmic DNA colocalized with AIM2. Control cells showed average colocalization fraction of 0.06, but in the treatment group it was 0.33. This was further confirmed by co-staining of cGAS protein, which is one of the DNA sensing proteins in the cytoplasm, with the DNA puncta in the cytoplasm of the macrophages stimulated with eCIRP (**Supplementary Figure S3**). We observed that the DNA released to cytoplasm was detected by cGAS whereas the nascent release of DNA from nucleus was not associated with cGAS yet.

### The nuclear DNA entering the cytoplasm is processed through autophagy pathway

**Figure 4:**
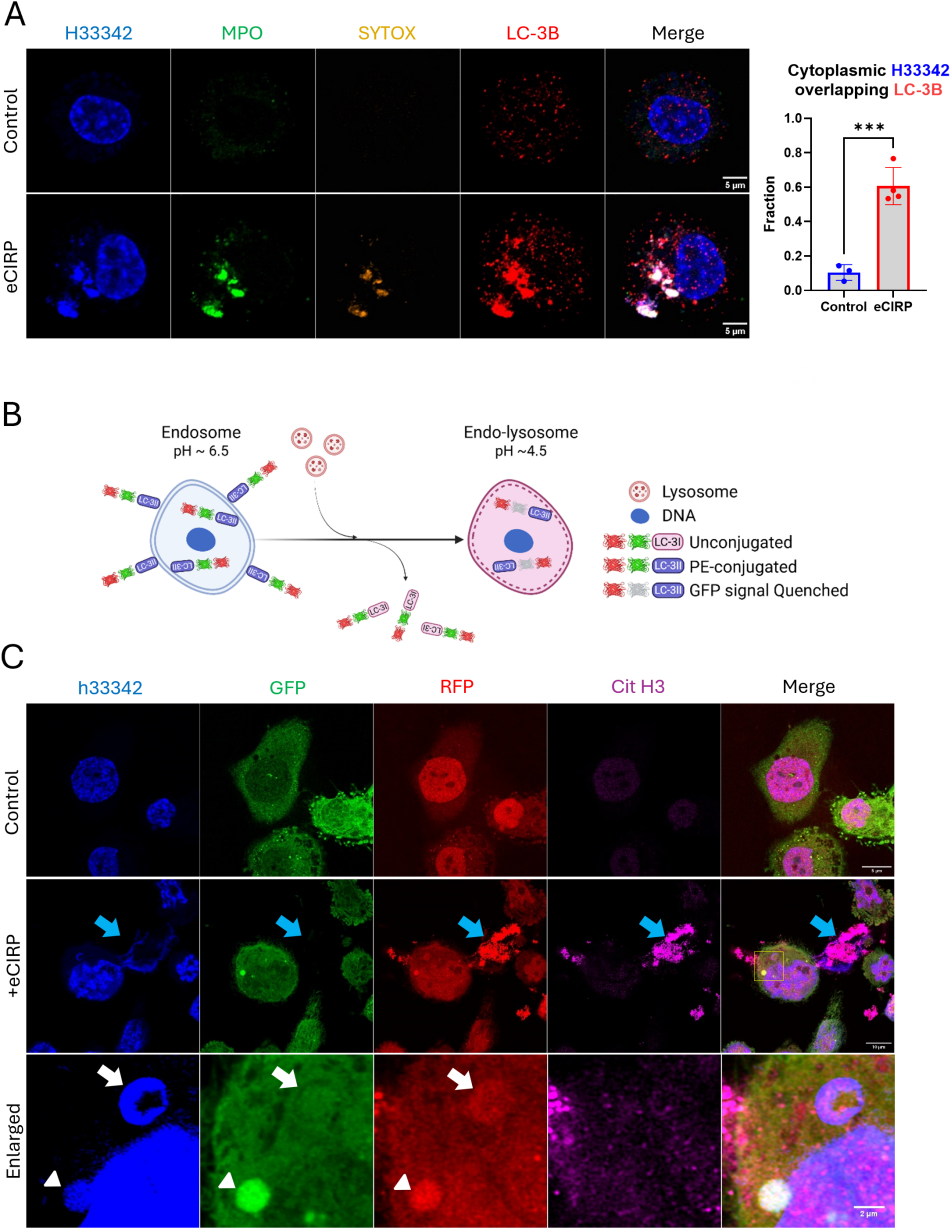
Cytoplasmic nuclear DNA is incorporated in autophagosome. The DNA released from the nucleus met LC3 and released together with LC3, **(A)** The cells treated with eCIRP showed significant accumulation of LC3 in the DNA puncta. The extracellular traps were also intensely stained with antibody against LC3B, arrow. Scale bar is 5 μm. For colocalization analysis of control cells (n = 3) and eCIRP-treated cells (n = 4), Manders’ coefficient was evaluated and showed significant colocalization of cytoplasmic DNA and LC-3B by eCIRP treatment. Student *t*-test: ***p < 0.001. The autophagy flux sensor RFP::GFP::LC3B was used in this study. The schema shows how the biosensor works in the cytoplasm of the macrophages, **(B)** The biosensor was used to show nuclear DNA puncta were trafficked to the lysosomal compartment in this study. Both RFP and GFP of the biosensor is fluorescent in neutral pH of the cytoplasm, however, when the biosensor is in acidic pH condition of endo-lysosomal lumen, the fluorescence of GFP is quenched while RFP is still fluorescent. As the pH of intra-luminal space of the endosome and endo-lysosome gradually decreases, thus, this biosensor is differentially fluorescent in terms of its subcellular localization in a cell. The cells were treated with eCIRP (2 μg/mL) for overnight. Confocal microscopy of the macrophages transduced with the biosensor gene visualized the DNA with both green and red fluorescence signal high, arrowhead, and red fluorescence only DNA punctum, white arrow, concomitantly in a cell, **(C)** METs released from the cell only have bright red color, blue arrow. Scale bars are 5 μm for control cell, 10 μm for eCIRP treated cell, and 2 μm for enlarge panel.

As we showed that the DNA puncta in the cytoplasm can be stained with SYTOX Orange, we posited that the DNA is incorporated into intracellular vesicular compartments and liberated to the outside of the cells. To investigate this, we tested the autophagy pathway because of its relevance for the clearance of undesired proteins and organelles. In macrophages stimulated with eCIRP, the nuclear DNA puncta strongly colocalized with LC3II (**Figure 4A**). Some of the nuclear DNA puncta stained exclusively with anti-LC3B antibody staining. This indicates LC3 assisted autophagosome formation preceded the DNA staining with SYTOX-Orange staining, which we speculated must happen in the endosomal compartment. The colocalization analysis showed a significant increase in the fraction of cytoplasmic DNA colocalized with LC3. Control cells showed average colocalization fraction of 0.10, but in treatment group it was 0.61. We also observed LC3 also colocalized with the METs released from the cells. To verify the presence of LC3 in the process of MET release and significant colocalization of intracellular LC3, we employed an autophagic flux biosensor, RFP::GFP::LC3B (**Figure 4B**). This biosensor can differentiate the autophagy processes due to the GFP instability in acidic environments. It was significantly located in the DNA puncta inside the cells and in the METs exposed to the extracellular environment (**Figure 4C**, **Supplementary Figure S5**). We could observe two distinctive DNA puncta in the same cell. One DNA punctum contained high levels of both colors, and the other contained only red fluorescence. It is speculated that the DNA in the cytoplasm was incorporated into the autophagosome which was high in both green and red fluorescence. Subsequent lysosomal fusion formed a phago-lysosomal vesicle whose acidic environment quenched the green fluorescence while remaining positive for red fluorescence. DNA released to the extracellular space had bright RFP fluorescence without GFP fluorescence, indicating that it had undergone lysosomal processing and exposure to its acidic pH environment prior to its release by the cells.

### Nuclear DNA phagophore containment is mediated by ESCRT machinery

**Figure 5:**
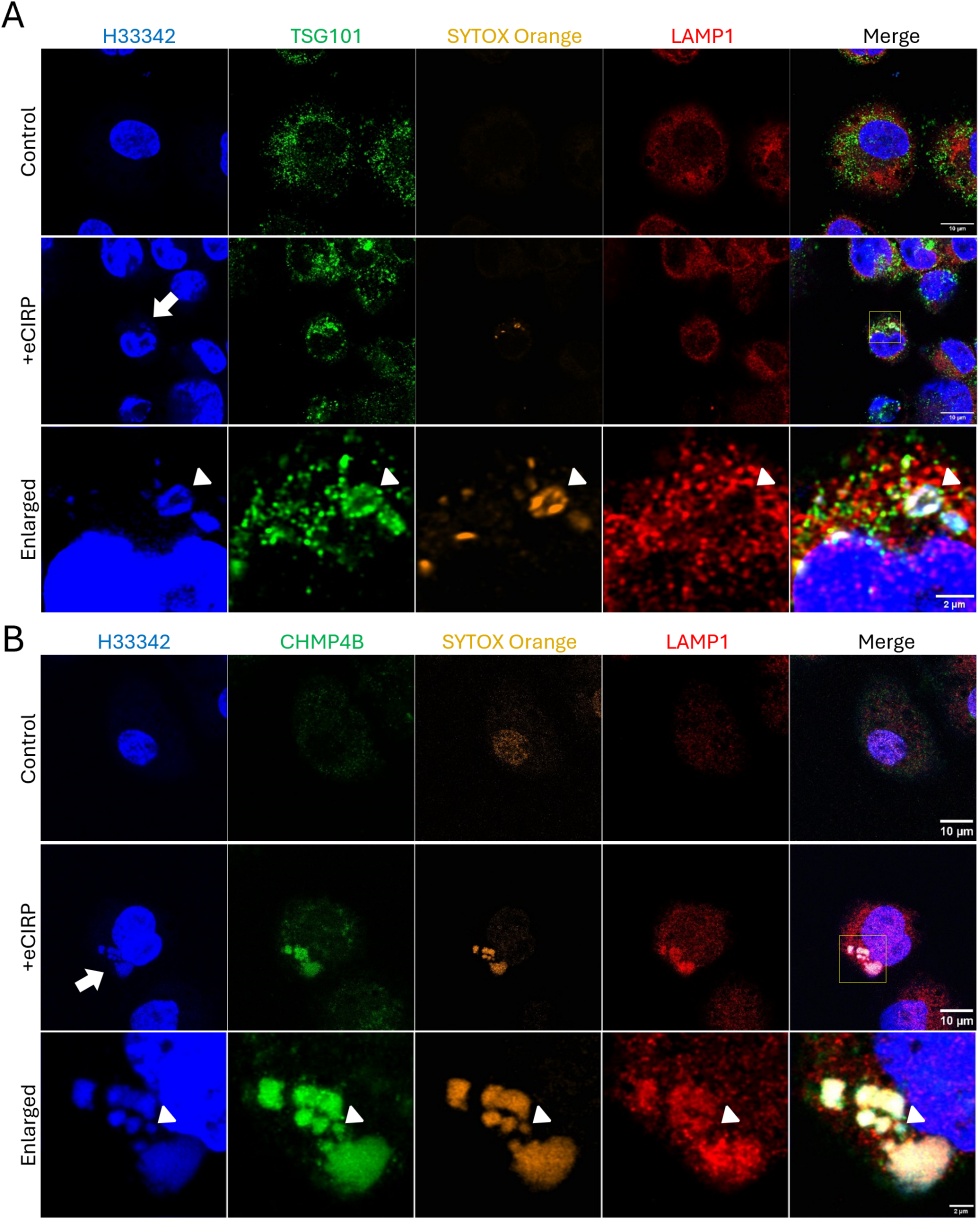
Autophagosome closure was mediated by ESCRT machinery. ESCRT machinery involved in the process of nuclear DNA puncta processes in the cytoplasm. TSG101 (ESCRT-I) was highly accumulated on the DNA puncta with SYTOX Orange dye, **(A)** The TSG101 image with eCIRP treatment was a representative image of 5 different cells. The DNA in the cytoplasm was intensely stained with anti-TSG101 antibody, arrow. LAMP-1 is not yet recruited to the DNA, arrowhead. The ESCRT-III complex protein CHMP4B was proven to be recruited to the DNA puncta in the cytoplasm and these DNA puncta are in the compartment positive to both SYTOX Orange and LAMP-1 Proteins. **(B)** The arrow indicates the DNA puncta was accumulated in the cytoplasm. LAMP-1 was highly accumulated in the puncta compartment. The arrowhead indicates one punctum is not positive to the LAMP-1 was not recruited. Scale bar is 10 μm for whole cell picture and 2 μm for the enlarged picture. The CHMP4B image with eCIRP treatment was a represent image of 3 different cells.

To evaluate how the free nuclear DNA entering the cytoplasm is membrane-isolated into a phagophore, the macrophages were further analyzed for colocalization with components of the Endosomal Sorting Complex Required for Transport (ESCRT) machinery. Specifically, we assessed TSG101 and CHMP4B, which are components of ESCRT-I and ESCRT-III complexes, respectively. We observed that the SYTOX Orange positive DNA puncta were brightly decorated with TSG101 (**Figure 5A**, bottom panel). It is noted that LAMP-1 was not detected in the DNA puncta (**Figure 5A**, bottom panel, arrowhead), indicating that lysosomal fusion had not yet happened. We also observed co-localization of SYTOX Orange positive DNA puncta with Antibody staining to CHMP4B (**Figure 5B**, middle and bottom panel). However, different from TSG101^+^ puncta, CHMP4B^+^ puncta stained bright for LAMP-1 (**Figure 5B**, middle and bottom panel), indicating lysosomal fusion or recruitment in close proximity to the DNA puncta via ESCRT III machinery. Some of the DNA puncta were positive to CHMP4B staining but not yet for LAMP-1 (**Figure 5B**, bottom panel, arrowhead), showing that ESCRT machinery processing preceded lysosomal fusion. Taken together, our data show that ESCRT choreographs the nuclear DNA phagophore containment within an isolating membrane to enter the endosomal compartment.

### Extracellular traps are secreted via the endo-lysosomal pathway

**Figure 6:**
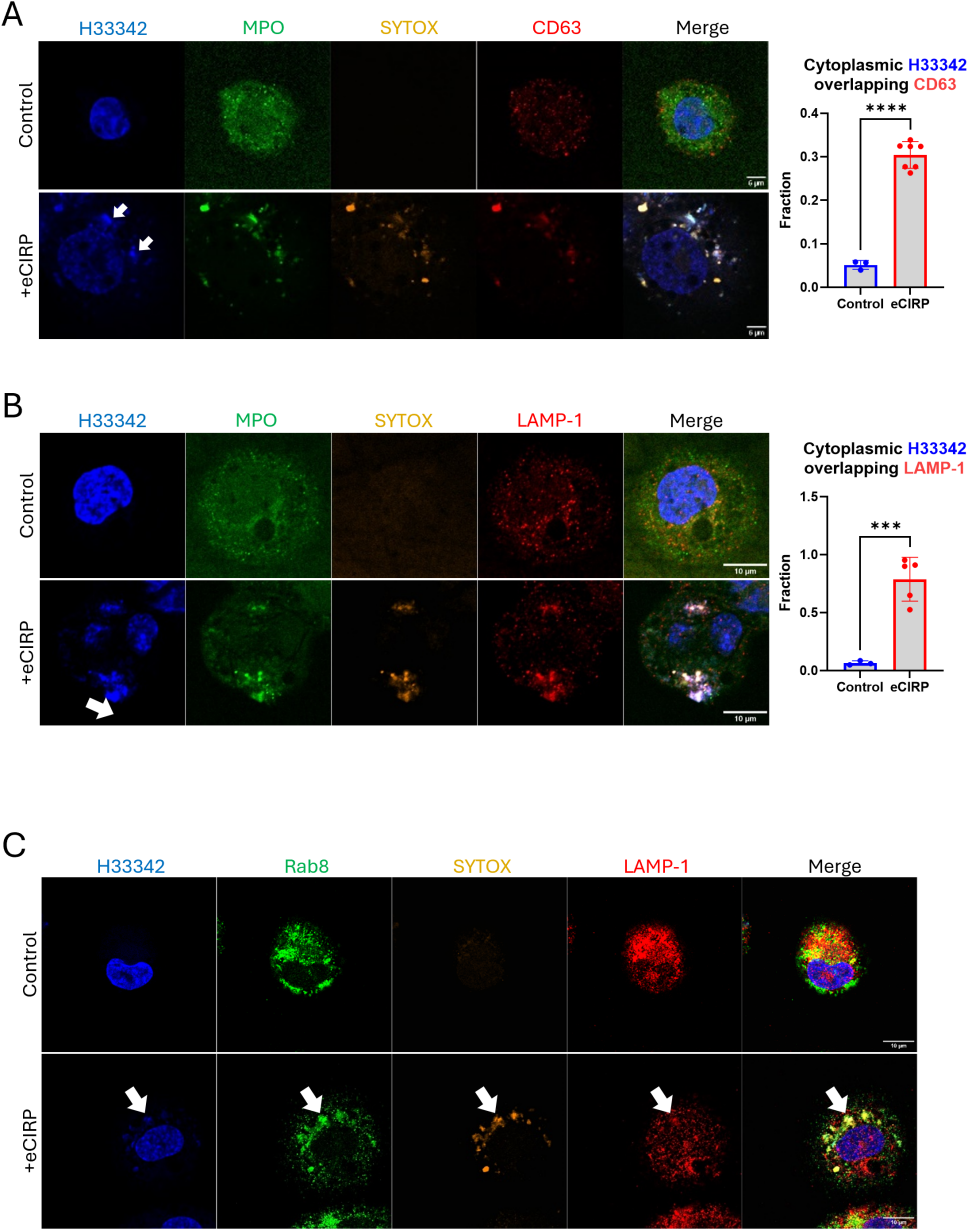
DNA was processed via endo-lysosomal trafficking. DNA released from nucleus was analyzed for lysosomal trafficking pathway. The DNA was analyzed with anit-CD63 antibody, **(A)** For colocalization analysis of control cells (n = 3) and eCIRP-treated cells (n = 7), Manders’ coefficient was evaluated and showed significant colocalization of cytoplasmic DNA and CD63 by eCIRP treatment. Student *t*-test: ****p < 0.0001. The immunofluorescence assay showed that majority of CD63 protein was associated to the DNA in the cytoplasm, arrows, whereas CD63 in the control cell showed random punctate distribution in the cytoplasm. Scale bar is 6 μm. LAMP1 is probed by the anti-LAMP1 antibody, **(B)** LAMP-1 is used as a pan-lysosomal marker and the localization of the lysosome was assessed by the antibody staining in the immunofluorescence assay. LAMP-1 is significantly co-localized with DNA, arrow. Scale bar is 10 μm. For colocalization analysis of control cells (n = 3) and eCIRP-treated cells (n = 5), Manders’ coefficient was evaluated and showed significant colocalization of cytoplasmic DNA and LAMP-1 by eCIRP treatment. Student *t*-test: ***p < 0.0001. Rab8 was used for secretory lysosomal marker in this study and accumulated significantly to the DNA release to cytoplasm of the cell treated with eCIRP. LAMP-1 showed co-localization to the Rab8 in the DNA puncta, **(C)** Scale bar is 10 μm. The Rab8 image with eCIRP treatment was a representative figure of 8 different cells.

We next characterized the role of the intracellular vesicular transport on the nuclear DNA release from the cytoplasm to the extracellular space through a series of immunofluorescence assays. We detected CD63 immunofluorescence on nuclear DNA puncta (**Figure 6A**, bottom panel). The colocalization analysis showed a significant increase in the fraction of cytoplasmic DNA colocalized with CD63. Control cells showed average colocalization fraction of 0.05, but in the treatment group it was 0.30. CD63 was used as an endosomal marker because not only it is expressed on the cell surface but is also abundantly present in late endosomes and lysosomes (18). Colocalization of CHMP7 and SYTOX Orange staining detected in the tip of nascent DNA released from the nucleus, which is still connected to the nucleus, (**Supplementary Figure S6**) indicates that the incorporation of the DNA into the endosomal compartment may be accompanied immediately while the DNA is still released from nucleus. Furthermore, we could observe that the DNA released was stained with antibody against lysobisphosphatidic acid (LBPA) and CHMP4B concomitantly (**Supplementary Figure S7**). LBPA is rich in late endosomes. It co-stained with DNA puncta, which were decorated with CHMP4B proteins. These data indicate that the DNA puncta was incorporated to the endosomal compartment. To show that the DNA puncta undergo secretory lysosomal processing we evaluated for colocalization with LAMP-1 and Rab8a, which showed significant enrichment in the DNA puncta. LAMP-1 is frequently used as a pan-lysosomal marker. Most of the LAMP-1 proteins were significantly enriched in the region of the nuclear DNA puncta (**Figure 6B**, bottom panel). The colocalization analysis showed a significant increase in the fraction of cytoplasmic DNA colocalized with LAMP-1. Control cells showed average colocalization fraction of 0.07, but in the treatment group it was 0.79. MPO and SYTOX staining were used to identify the DNA puncta released. Immunofluorescence of Rab8a showed that the protein localized at the nuclear DNA puncta (**Figure 6C**, bottom panel). Rab8a proteins were evenly distributed around massive DNA puncta, which is clearly distinguished from the Rab8a positive small vesicles in the cytoplasm of control cell. Rab8a is a protein that regulates the fusion of secretory vesicles with the plasma membrane. Rab8a has been shown to participate in the unconventional secretion of the proinflammatory cytokine IL-1β (19). These observations indicate that the DNA puncta, packaged in vesicles via autophagic flux, were destined to endo-lysosomal processing.

### Vital release of METs requires acidic processing in the lysosomal compartment

**Figure 7:**
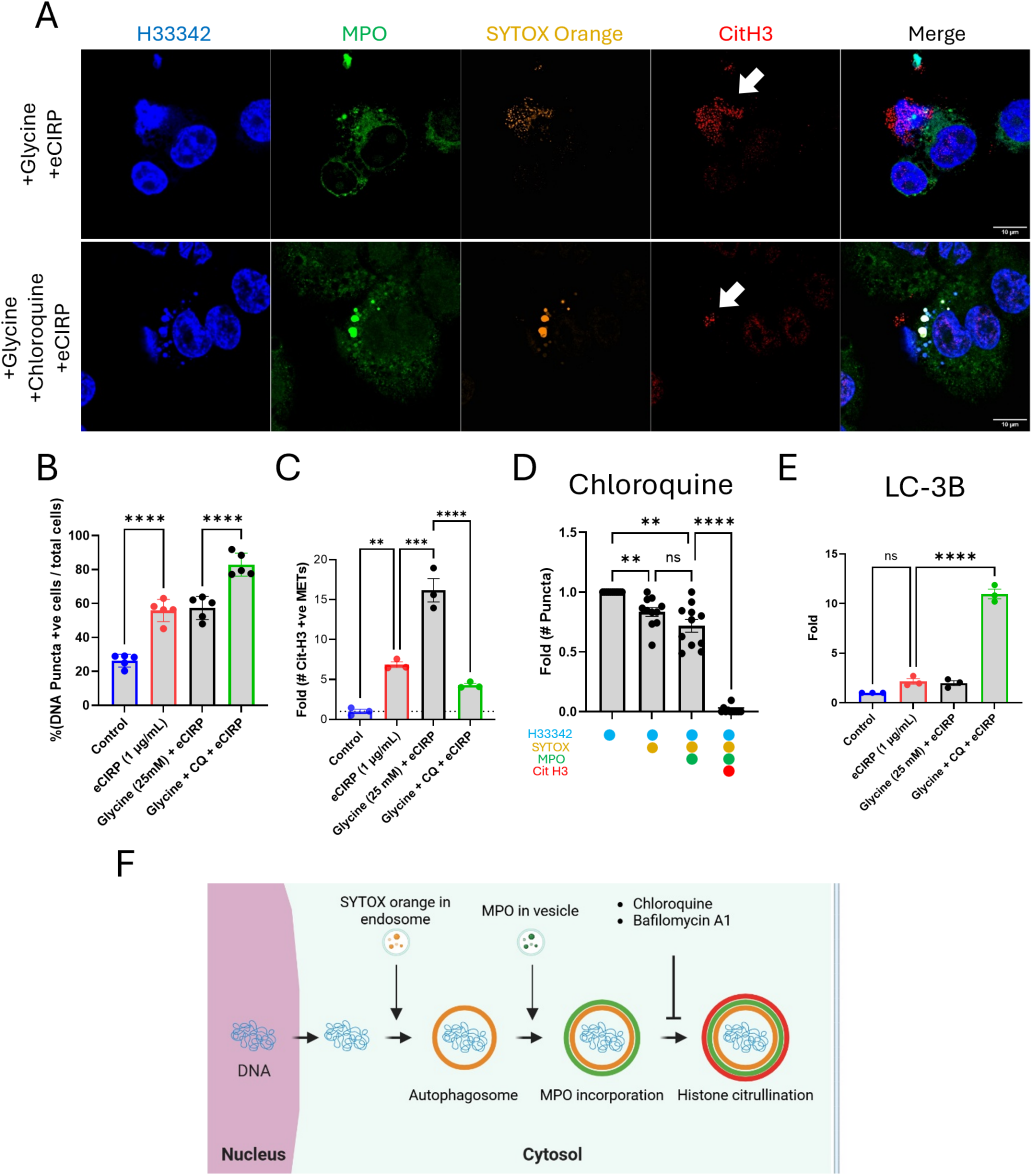
Extracellular traps release was inhibited by Chloroquine. The treatment of chloroquine to the macrophages was analyzed by immunofluorescence assay using anti-MPO and Cit H3 antibodies, **(A)** Chloroquine significantly reduced the level of citrullination in the DNA puncta while the MPO is still persistent, arrow. Scale bars are 10 μm. The percentage of DNA puncta positive cells in the microscopic field was calculated, **(B)** DNA puncta in each cell was detected by the SYTOX Orange staining image. One-way ANOVA: **** < 0.0001.The quantitative analysis of the number of Cit H3 positive METs was done with low magnification image analysis with chloroquine (CQ) treatment, **(C)** One-way ANOVA: ** < 0.01, *** < 0.001, and **** < 0.0001. The analysis of DNA puncta in the cytoplasm of the macrophages treated with chloroquine showed the lack of citrullination of Histone 3 protein (N = 11 cells), **(D)** One-way ANOVA: not significant, ** < 0.01, and **** < 0.0001. The effect of chloroquine on the macrophages was confirmed by the western blot of LC3B in the cytoplasm. LC3B was significantly accumulated in the cytoplasm by the treatment of chloroquine in the macrophages, **(E)** The data was obtained by analyzing three biologically independent experiments. One-way ANOVA: ns, not significant and **** < 0.0001. Overall process of the DNA puncta in the cytoplasm was summarized in the schematic diagram, **(F)**.

To show the role of lysosomes in the processing of extracellular traps release in live cells we have employed the lysosomotrophic agent, chloroquine. Chloroquine is a weak base that accumulates in lysosomes as a protonated form, which increases the pH of the lysosome (20). Chloroquine is considered an anti-inflammatory medication and is used to treat autoimmune diseases like rheumatoid arthritis, systemic lupus erythematosus (SLE), and sarcoidosis, due to its ability to suppress the immune system and reduce the production of inflammatory cytokines (21). As a result of chloroquine treatment, DNA puncta significantly accumulated in the cytoplasm (**Figure 7A**, middle panel). The resulting extracellular traps were decreased compared to the cell treated with eCIRP alone (**Figure 7A**, arrow). The puncta were bigger in size and densely packed with SYTOX Orange and anti-MPO antibody than the DNA puncta observed in the cells treated with only eCIRP. Quantitative analysis of large, stitched high resolution images showed accumulation of DNA puncta, positive to SYTOX Orange staining, in cells stimulated with eCIRP, which was further increased by the addition of chloroquine (**Figure 7B**). While there was significant accumulation of DNA in the cytoplasm from high resolution confocal images quantitative analysis from low magnification confocal images showed significant decrease in the CitH3 positive extracellular DNA compared to that of eCIRP alone treated (**Figure 7C**). Furthermore, we couldn’t observe many of the DNA puncta positive to CitH3 in the cells treated with chloroquine (**Figure 7D**). Western blot analysis of LC3B further supported the effect of chloroquine on the accumulation of the DNA puncta in the cytoplasm (**Figure 7E**, **Supplementary Figure S9**). We have observed significant accumulation of LC3 in the cytoplasm by treatment of chloroquine. The LC3 level was increased 10 times higher than the untreated cells. Taken together, we have shown that the acidic environment in the lysosomes were critically required for the histone citrullination and MET release processes (**Figure 7F**).

## Discussion

METs have been identified as an essential defense mechanism against bacterial infection. However, the dysregulation of METs is associated with different diseases including inflammation and autoimmune disorder where METs can cause tissue damage by activating the host defense and immune mediator production (22). METs have also been implicated in cancer progression and metastasis (23). Previously, we observed that the treatment of eCIRP-triggered TLR4 receptor activation. Downstream signaling propagated to activate GSDMD and resulted in pyroptotic cell death and massive extracellular traps were released (6). Here, we identify MET release from live macrophages and elucidate, for the first time, the major molecular and subcellular mechanisms involved in the vital release of METs.

GSDMD is a protein that plays a critical role in pyroptosis, a form of inflammatory cell death. The primary function of GSDMD is to form pores in the plasma membrane, leading to the release of pro-inflammatory cytokines like IL-1β and IL-18, cell swelling, and membrane rupture (24). GSDMD contains two main domains: the N-terminal domain (GSDMD-NT) and the C-terminal domain (GSDMD-C) connected by a linker region (25). Upon proteolytic cleavage by activated caspase-1 as part of inflammasome activation, GSDMD-NT oligomerizes and forms cell membrane pores. GSDMD pore formation has been reported not only on the plasma membrane (26) (27, 28), but also on a number of membra-bound organelles (24, 29–32). While GSDMD targets the plasma and organelle membranes, some studies suggest it can also affect the nuclear membrane, causing nuclear swelling and potentially disrupting nuclear integrity (33, 34). We have previously observed nuclear swelling in the process of pyroptosis induced by eCIRP (6). It seemed that the swelling was coordinated with the plasma membrane rupture because SYTOX Orange staining of the cell happened at the same time as nucleus swelling. Thus, we hypothesized that GSDMD could be responsible for initiating the nuclear DNA release in the live macrophages. Indeed, we were able to obtain direct evidence that nuclear DNA enters the cytoplasm via GSDMD pores. While we showed accumulation of GSDMD-NT in the nuclear fraction, we also showed that most of GSDMD-NT accompanied the nuclear DNA released to the cytoplasm, suggesting that GSDMD pores are quickly eliminated by a yet undetermined active process in the cells. This assumption is backed by the co-localization of CHMP7 to the nuclear DNA released into the cytoplasm (**Supplementary Figure S6**). CHMP7 is part of the ESCRT-III complex within the ESCRT machinery. It plays a crucial role in the recruitment and assembly of ESCRT-III at the nuclear envelope, particularly during nuclear envelope reformation and repair (35). In this work, we could detect CHMP7 proteins in the DNA puncta and the accumulation of CHMP7 around the DNA puncta. We could find that the CHMP7 proteins localize in proximity of nascent DNA release, where we can find the signal of SYTOX Orange. Further analysis suggests a role for ESCRT machinery in processing the DNA, as LBPA, TSG101, and CHMP4B proteins co-stained with SYTOX Orange in the DNA puncta. Interestingly, CHMP4B was accompanied with LAMP-1 proteins, which are pan-lysosomal marker while LBPA and TSG101 are not. Therefore, we concluded that ESCRT machinery participates in various processes including nuclear envelope repair, nuclear DNA vesicle formation, and fusion to other intracellular vesicles (35–37).

SYTOX dead cell staining dye was designed for detecting dead cells by staining intracellular DNA exposed due to plasma membrane rupture. We initially used it for the purpose of staining METs released outside of the cells. Considering the impermeability of SYTOX dye for live cells, it was unusual that we found intracellular puncta brightly stained with SYTOX Orange in the immunofluorescence staining, which is significantly brighter than extracellular DNA stained. Micronuclei in the control macrophages were negative to SYTOX dye (data not shown). However, we concluded that the SYTOX Orange dye was internalized by the cells via endocytosis and transported through the endosomal compartment. This is supported by the fact that, while we could find SYTOX Orange positive DNA puncta in the cytosol, the nuclear DNA remained negative to SYTOX Orange dye. This observation enabled us to explain the vesicular incorporation of cytosolic DNA in live cells. Therefore, selective staining of vesicular DNA with SYTOX Orange dye is the key aspect of vital METosis in our study. We first speculated that the staining may happen because of the mitochondrial DNA in an endo-lysosomal compartment of the cell where the endocytosed SYTOX Orange dyes meet the DNA in the autophagosome. However, the DNA staining with MPO and CitH3 immunostaining indicated that this DNA originated from the nucleus. The DNA released from the live macrophages was incorporated with MPO and had histone modification, citrullination. As it has been previously reported, the METs released from macrophages by pyroptotic cell death showed MPO association and histone modification by protein arginine deiminase (PAD) in the cells (38, 39). Citrullinated H3 (CitH3) is widely used as a surrogate marker for detecting extracellular trap DNA in combination with MPO (40). We showed that this extracellular DNA also has MPO and CitH3 positive. Even some of the DNA puncta in the cytoplasm were also differentially stained with anti-MPO and anti-citrullinated Histone 3 antibodies (**Figures 1A, B**). While no significant signal was detected in the nucleus, each DNA puncta showed both MPO and CitH3 or only single staining, or none of them. Unlike the conventional model of METs, which MPO and PAD4 are translocated to nucleus, however, in vital MET formation MPO is not translocated to the nucleus but rather to each individual nuclear DNA vesicle in the cytoplasm, and they are mutually exclusive. Based on our data, MPO incorporation to DNA was done in the vesicular compartment because the nascent DNA released was not immediately associated with MPO. Furthermore, histone citrullination was also observed in only limited number of DNA puncta, whereas there is no significant hyper-citrullination in any nucleus we have observed. There may be a stepwise maturation process of the METs prior to their release to the extracellular space.

As shown in **Figure 1**, vital METs have the same MPO and CitH3 modifications as the pyroptotic METs. We have not yet found any marker allowing to differentiate vital METs from pyroptotic METs. The best approach we have identified was to suppress pyroptosis at the very end of the pathway, which is the plasma membrane rupture and release of the whole nuclear DNA. It was recently known that glycine effectively inhibits NINJ1 pore formation, which is the direct cause of plasma membrane rupture (14). According to the analysis of the shape and fluorescence intensity of nuclei, we observed about 4% of the cell nuclei in glycine treated sample have shrunk nuclear morphology and were significantly bright (**Figure 2D**). Considering this population was 2% in the cells treated with eCIRP alone, there were only 2% increase in apoptotic cell death in the presence of glycine. This means the glycine treatment effectively suppressed the onset of cell death. It showed that glycine treatment effectively prevents cell rupture from the treatment of eCIRP. The increased number of the cells releasing METs (**Figure 2F**) suggest that the cells survived from the membrane rupture may contribute to the net increase of the METs through vital release processes.

The detection of the intracellular DNA is mediated by multiple proteins in the cytoplasm, so called intracellular DNA sensor including cGAS and AIM-2 (41) (42) (43) (44). Then the DNA detected triggers inflammation response in the cells and eventually eliminated by intracellular process including enzymatic degradation, autophagy, and exocytosis. The extravesicular secretion of DNA was described by many researchers from different species (45). Yokoi et al. identified the Micronuclei was packaged into the multivesicular bodies (MVBs) in the tetraspanin (CD63)-based endosomal sorting pathway and released into extracellular space in the form of exosomes (46). In this study we showed that the nuclear DNA in the cytoplasm was packaged into CD63 positive vesicles. Since this process could be assisted by the ESCRT pathway (37) as we showed that TSG101, CHMP4B, and CHMP7 were detected from the nuclear DNA puncta in the cytoplasm, thus, we don’t exclude the possibility of exosome secretion of the DNA puncta from the macrophages.

The extracellular release process of the nuclear DNA showed LC3B lipidation on the DNA puncta and massive secretion of LC3 with DNA in the macrophages. This resembles what was observed from mast cell degranulation (47). It was reported that LC3-II co-localizes with a secretory lysosomal marker, CD63, and released extracellularly along with degranulation in mouse bone marrow derived mast cells. In the macrophages in this study, the accumulation of LC3 in the secretary vesicles was observed in the cells treated with chloroquine, which elevates pH in the lumen of lysosomes and impairs the function of lysosome. This was shown by the western blot analysis of cell lysate from the cells co-treated with Glycine, Chloroquine, and eCIRP. Chloroquine caused significant reduction of CitH3 staining in the quantification of the immunofluorescence in this work. Thus, we reached to the conclusion that the DNA was not efficiently secreted through the lysosomal secretion pathway.

The intracellular DNA puncta analysis under the influence of chloroquine suggested that the citrullination in the Histone 3 protein was very poor in the cytoplasm. PADs are cytoplasmic enzymes, and there are five isoenzymes identified in human (48). Among them, the expression of PAD2 and PAD4 in macrophages was reported (49) (50). It has been thought that PAD4 meets histone proteins in the nucleus and citrullinates them because it has nuclear localization signal (38)0. However, we couldn’t observe any significant citrullination in the nucleus from the live cells, while the DNA puncta have intense staining of CitH3 by immunofluorescence staining. We observed that the treatment of chloroquine resulted in significant decrease of the citrullination in the DNA puncta. As it is well known that chloroquine was known to inhibit proteolytic enzymes in lysosome with the increase of 0.5 to 1.0 pH unit from pH 4.5 in lysosome (20) (51) (52). Since PAD2 and 4 still have enzymatic activity at mild acidic pH 6.4 (53), we haven’t expected such significant reduction in citrullination on Histone proteins in the lysosomes under the influence of chloroquine. Similar effects with the treatment of bafilomycin A1, which inhibits lysosomal V-ATPase, were obtained (**Supplementary Figure S10**). This suggests that chromatin modification by proteolytic enzymes should occur prior to the enzyme reaction of PADs in lysosomes. The relaxed chromatin structure could give more access to PADs to catalyze citrullination on histone proteins. Considering all the circumstances we discuss here; we speculated that the assistance of proteolytic enzymes in lysosome promoted relaxation of the chromatin structure of the nuclear DNA and exposed the arginine residues on histones in the nucleosomes to PADs in lysosome.

Vital release of extracellular traps has been observed in neutrophils previously (7–9). Vital NETosis requires vesicular trafficking of DNA from the nucleus to the extracellular space. Vesicles of DNA budded from the nuclear envelope, passed through the cytoplasm, and coalesced with the plasma membrane, thereby delivering the NET out of the cell without requiring membrane perforation. The vital release of macrophages in this study has shown similarity in the way of DNA release processes to the vital NETosis.

Our work elucidates several questions, but it also opens many more. The speculation that vital METosis is more inflammatory than pyroptotic METosis remains to be verified. We also still don’t know whether the nuclear DNA used to produce vital METs is selectively enriched for certain chromosomal regions, nor whether the gene content of the released METs plays any role in cell-to-cell communication. Neither is known how the release of vital METs influences macrophage manyfold activities, including degranulation, phagocytosis, T cell regulation, and foreign body reaction. We hope this study will provide a solid foundation for future investigations of these and other question, such as the precise role of vital METs in health and disease.

In conclusion, we have demonstrated that the release of nuclear DNA is mediated by GSDMD pores formed on nuclear envelope, and that nascent METs are secreted through autophagic and lysosomal processing. Vital METs may play an important role in immune defense by capturing and eliminating bacteria, as well as deleterious roles in tissue damage, inflammation, autoimmunity and cancer.

## Data Availability

The raw data supporting the conclusions of this article will be made available by the authors, without undue reservation.

## Supplementary material

Supplementary Figure 1 eCIRP induces DNA release from live THP-1 cells. The release of METs from live THP-1 macrophages treated with eCIRP (1 μg/mL) were observed by confocal microscopy. Single slice of confocal z-stack images was processed for seven different cells. Scale bar is 10 μm in all images.

Supplementary Figure 2 Nuclear condensation analysis by dot plot. The metadata from the nuclear confocal images were analyzed by FlowJo software. Mean fluorescence of nuclei was analyzed with nuclear area by dot plot. The rectangular gate was applied to the plot area where represents MFI high and small nuclear area. The count was compared to the total number of the nuclei in the microscopic field and summarized in **Figure 2D**.

Supplementary Figure 3 DNA release is associated with cGAS. Nuclear DNA released to cytoplasm was further analyzed by immunofluorescence assay with anti-cGAS antibody. The cell treated with eCIRP for overnight was fixed and immunostained with the antibody. The extended focus image of the cell (top panel) showed that DNA released was highly stained with cGAS. cGAS was detected with the cytosolic DNA. The DNA in the vesicles were co-stained with SYTOX Orange dye. Single z slice image (second row panel) was selected to show the nascent DNA release from the nucleus. The blown-up image of the yellow box (third row panel) showed the nascent DNA release from the nucleus and marked disconnection of the nuclear lamina, which visualized with the Lamin A/C immunostaining, white arrowhead. Unlike the DNA in the vesicle (arrow in the middle panel), the DNA in the middle of the release was negative to either of cGAS or SYTOX Orange. 3D projection of enlarged image (bottom panel) showed the protrusion of the DNA from the nucleus from 4 different x, y, z coordination, red arrowhead. Scale bar is 10 μm.

Supplementary Figure 4 western blot analysis of nuclear and cytosolic fraction of the cells. The western blot analysis for the nuclear fractions and cytoplasmic fractions were performed. Each fraction was probed with anti-Asp275 GSDMD-NT, anti-Lamin A/C, and anti-GAPDH antibodies. Each set of data was prepared from three biologically independent experiments.

Supplementary Figure 5 Confocal microscopy of RFP::GFP::LC3B and immunofluorescence of Cit H3. The macrophage transduced with RFP::GFP::LC3B was assay by immunofluorescence with anti-Cit Histone3 antibody. METs released from the cell showed intense RFP fluorescence, arrow. The enlarged image showed the cytoplasmic nuclear DNA puncta positive to both of green and red fluorescence. Scale bars are 10 and 1 μm for whole cell and blown up, respectively.

Supplementary Figure 6 DNA release process is accompanied with CHMP7 recruitment. CHMP7 immunofluorescence assay showed that the protein was significantly accumulated in the DNA puncta or proximity to the DNA released, arrow. The blown-up image showed that CHMP7 was recruited to the proximity to the nascent protrusion of nuclear DNA, arrowhead. Notable observation was that the tip of the DNA protrusion had overlapped signals of CMHMP7, SYTOX Orange and LAMP-1 altogether. Scale bars are 10 and 2 μm for whole cell image and enlarged image, respectively. The CHMP7 image with eCIRP treatment was a representative image of 4 different cells.

Supplementary Figure 7 Nuclear DNA went through endosomal trafficking. The endosomal marker, lyso-bis-phosphatidic acid (LBPA), was probed by immunofluorescence assay with anti-LBPA antibody. The cell was also stained with anti-CHMP4B antibody simultaneously. Both LBPA and CHMP4B are significantly accumulated where the DNA released from nucleus. The DNA puncta in the enlarged image showed that LBPA signal was in the DNA puncta and CHMP4B decorated the surrounding of the DNA puncta, arrowhead. Scale bars are 10 and 2 μm for whole cell and blown up, respectively. The LBPA image with eCIRP treatment was a representative image of 2 different cells.

Supplementary Figure 8 Nuclear DNA was significantly accumulated in the cytosol due to the dysfunction of Lysosome by Chloroquine treatment. The treatment of chloroquine (final concentration: 20 μM) to the macrophages was analyzed by immunofluorescence assay using anti-MPO and Cit H3 antibodies. Chloroquine significantly reduced the level of citrullination in the DNA puncta while the MPO is still persistent, arrow. Scale bars are 10 μm.

Supplementary Figure 9 LC3B western blot. LC3B level in the cytoplasm was measured by the western blotting with anti-LC3B antibody, which was used for the immunofluorescence assay in **Figure 4A**. Sample sets were prepared from three biologically independent experiments.

Supplementary Figure 10 Extracellular traps release was also inhibited by bafilomycin A1. The treatment of Bafilomycin A1 (final concentration: 20 nM) to the macrophages was analyzed by immunofluorescence assay using anti-MPO and Cit H3 antibodies, **(A)**. Bafilomycin A1 significantly reduced the level of citrullination in the DNA puncta while the MPO is still persistent, arrow. The quantitative analysis was done with low magnification image analysis with bafilomycin A1 treatment, **(B)**. One-way ANOVA: ** < 0.01 and **** < 0.0001. The analysis of DNA puncta in the cytoplasm of the macrophages treated with bafilomycin A1 showed the lack of citrullination of Histone 3 protein, **(C)**. One-way ANOVA: ns, not significant and **** < 0.0001.

Supplementary Figure 11 Normal distribution test of one way ANOVA analysis. Normal distribution of data for one-way ANOVA analysis was verified by quantile-quantile (QQ) plots and all the analysis showed normal distribution.

Movie S1 Time-lapse live imaging. The time-lapse microscopy was performed with SYTOX Orange staining. Cells were treated with eCIRP (1 μg/mL) with or without glycine. The time interval of imaging was 15 minutes. The experiment was performed for 16 h. Glycine was treated to the cells 30 minutes prior to the treatment of eCIRP. The time-lapse imaging was started immediately after eCIRP treatment. Red pseudo color was used for the fluorescence signal for SYTOX Orange dye, which dead cell nuclear DNA staining.

Movie S2 3 D reconstruction of Confocal z stack image for GSDMD-NT immunofluorescence. The confocal z stack images showed in **Figure 3A** were used for the 3-D reconstruction. 3-D volume view and animation was generated with Imaris software. Scale bar is 5 μm.
