# Supplementary figures and images for: Molecular and subcellular mechanisms of vital macrophage extracellular trap formation
Source: Front Immunol. 2025 Jul 31;16:1608428. doi: 10.3389/fimmu.2025.1608428 (PMC12350110; doi:10.3389/fimmu.2025.1608428)

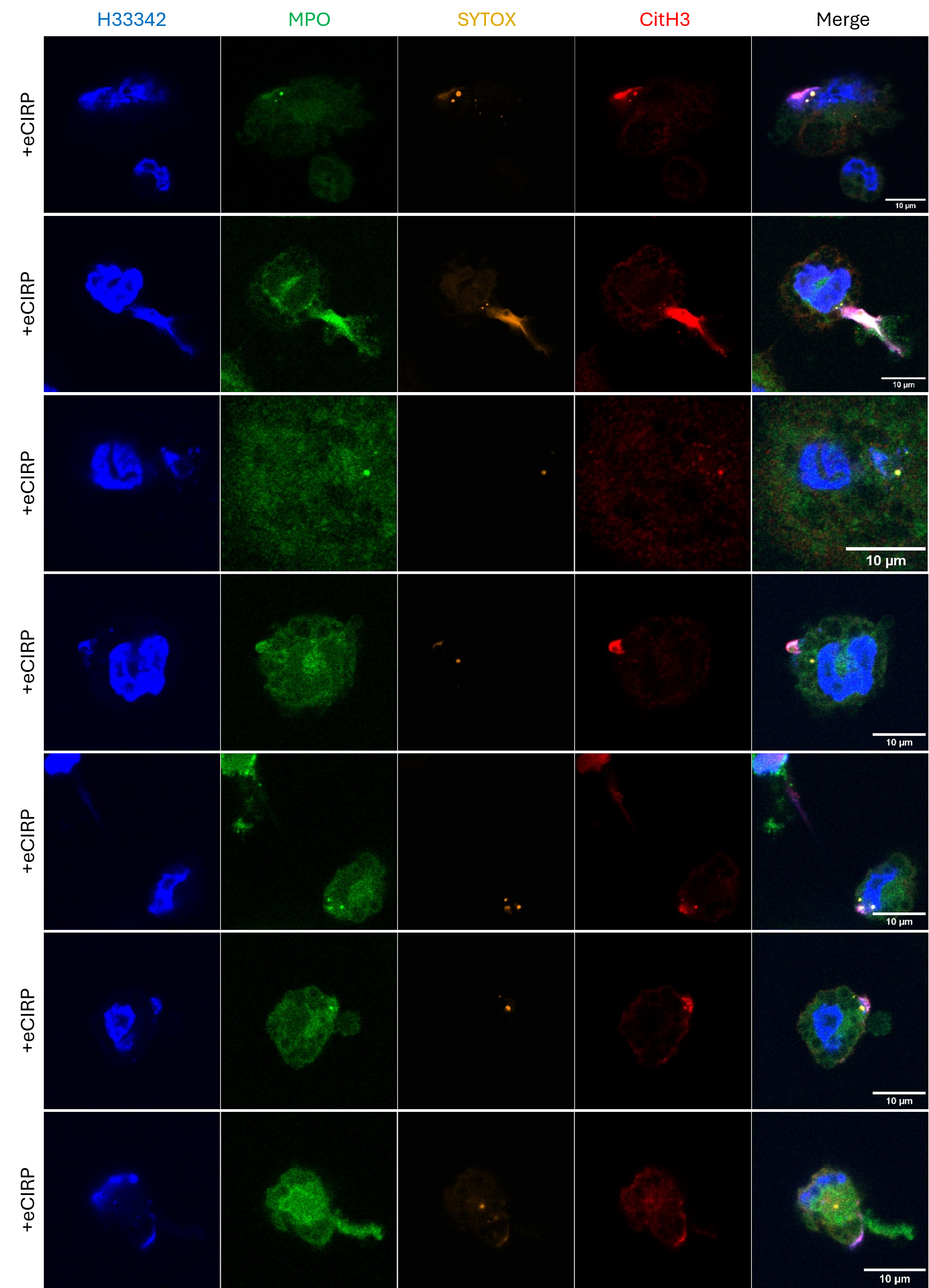

Supplement: Supplementary Figure 1 — eCIRP induces DNA release from live THP-1 cells. The release of METs from live THP-1 macrophages treated with eCIRP (1 μg/mL) were observed by confocal microscopy. Single slice of confocal z-stack images was processed for seven different cells. Scale bar is 10 μm in all images. [file Image1.tif]

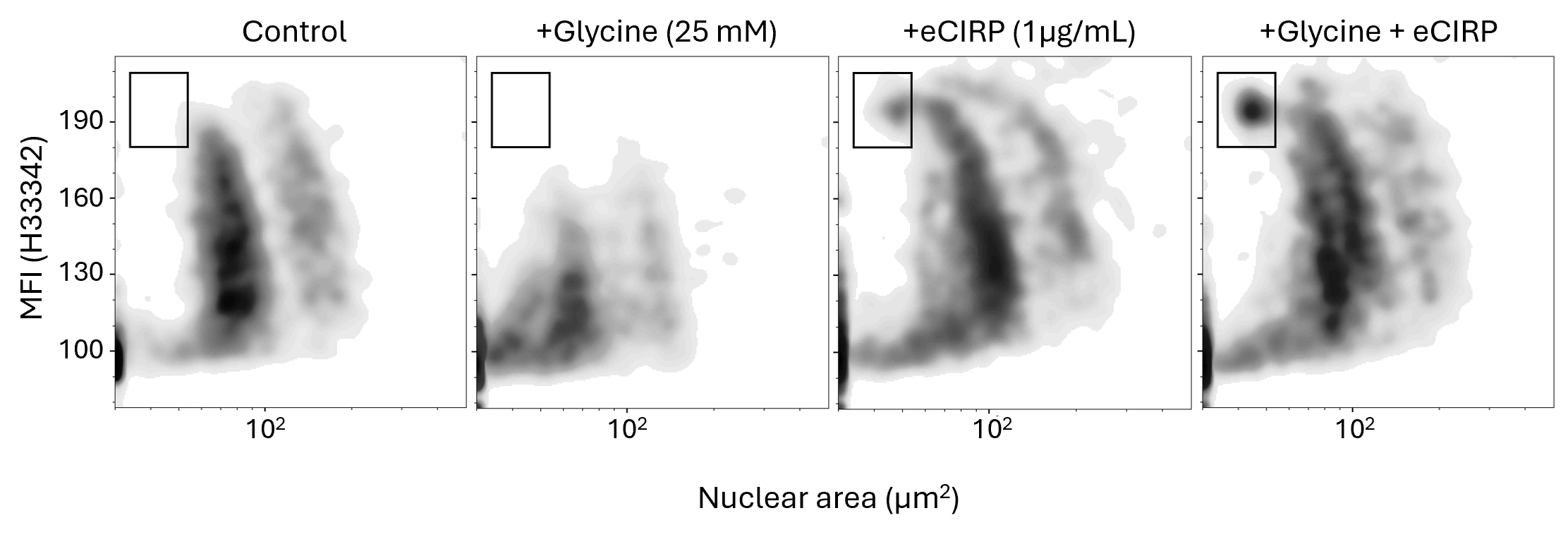

Supplement: Supplementary Figure 2 — Nuclear condensation analysis by dot plot. The metadata from the nuclear confocal images were analyzed by FlowJo software. Mean fluorescence of nuclei was analyzed with nuclear area by dot plot. The rectangular gate was applied to the plot area where represents MFI high and small nuclear area. The count was compared to the total number of the nuclei in the microscopic field and summarized in Figure 2D . [file Image2.tif]

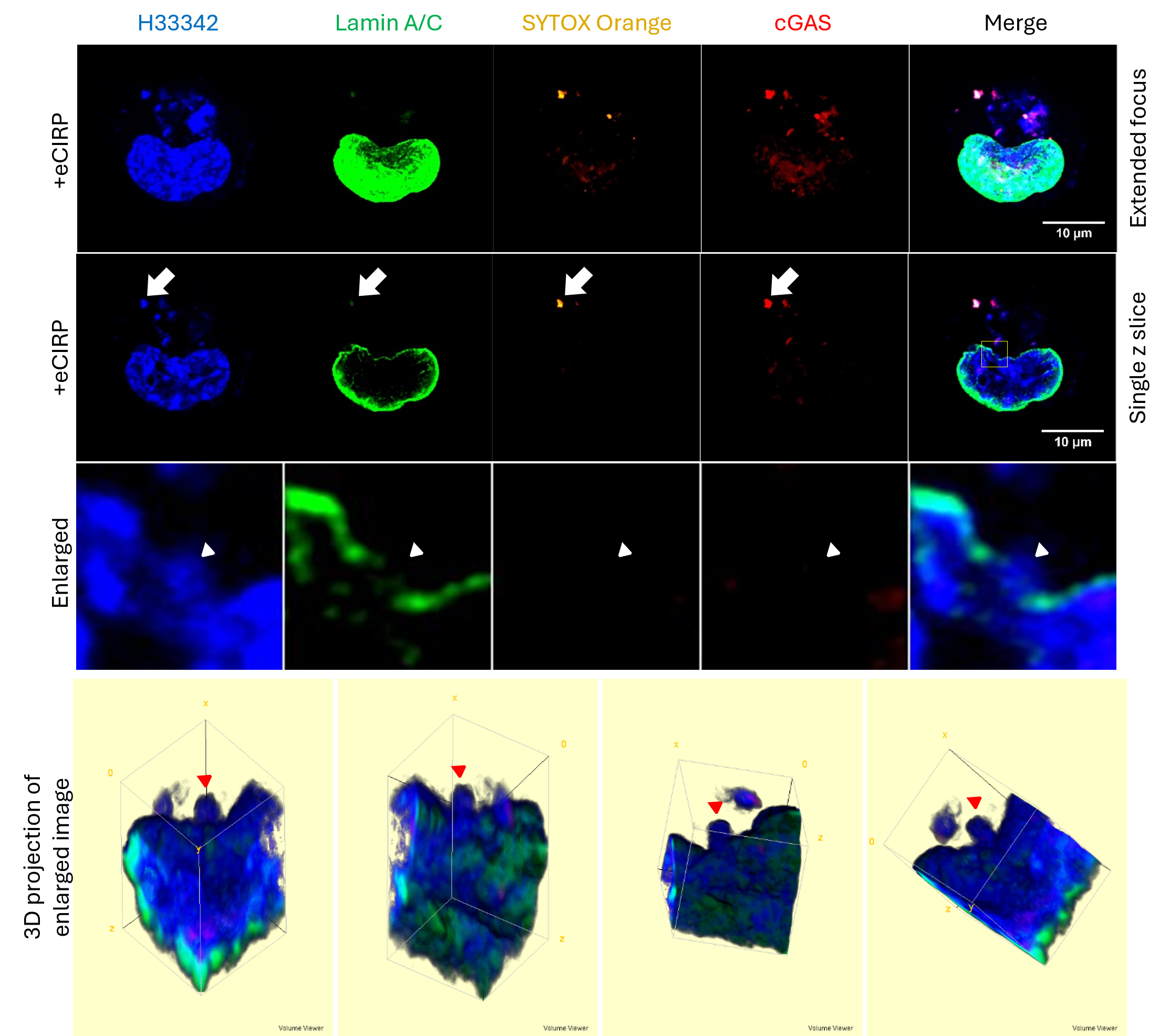

Supplement: Supplementary Figure 3 — DNA release is associated with cGAS. Nuclear DNA released to cytoplasm was further analyzed by immunofluorescence assay with anti-cGAS antibody. The cell treated with eCIRP for overnight was fixed and immunostained with the antibody. The extended focus image of the cell (top panel) showed that DNA released was highly stained with cGAS. cGAS was detected with the cytosolic DNA. The DNA in the vesicles were co-stained with SYTOX Orange dye. Single z slice image (second row panel) was selected to show the nascent DNA release from the nucleus. The blown-up image of the yellow box (third row panel) showed the nascent DNA release from the nucleus and marked disconnection of the nuclear lamina, which visualized with the Lamin A/C immunostaining, white arrowhead. Unlike the DNA in the vesicle (arrow in the middle panel), the DNA in the middle of the release was negative to either of cGAS or SYTOX Orange. 3D projection of enlarged image (bottom panel) showed the protrusion of the DNA from the nucleus from 4 different x, y, z coordination, red arrowhead. Scale bar is 10 μm. [file Image3.tif]

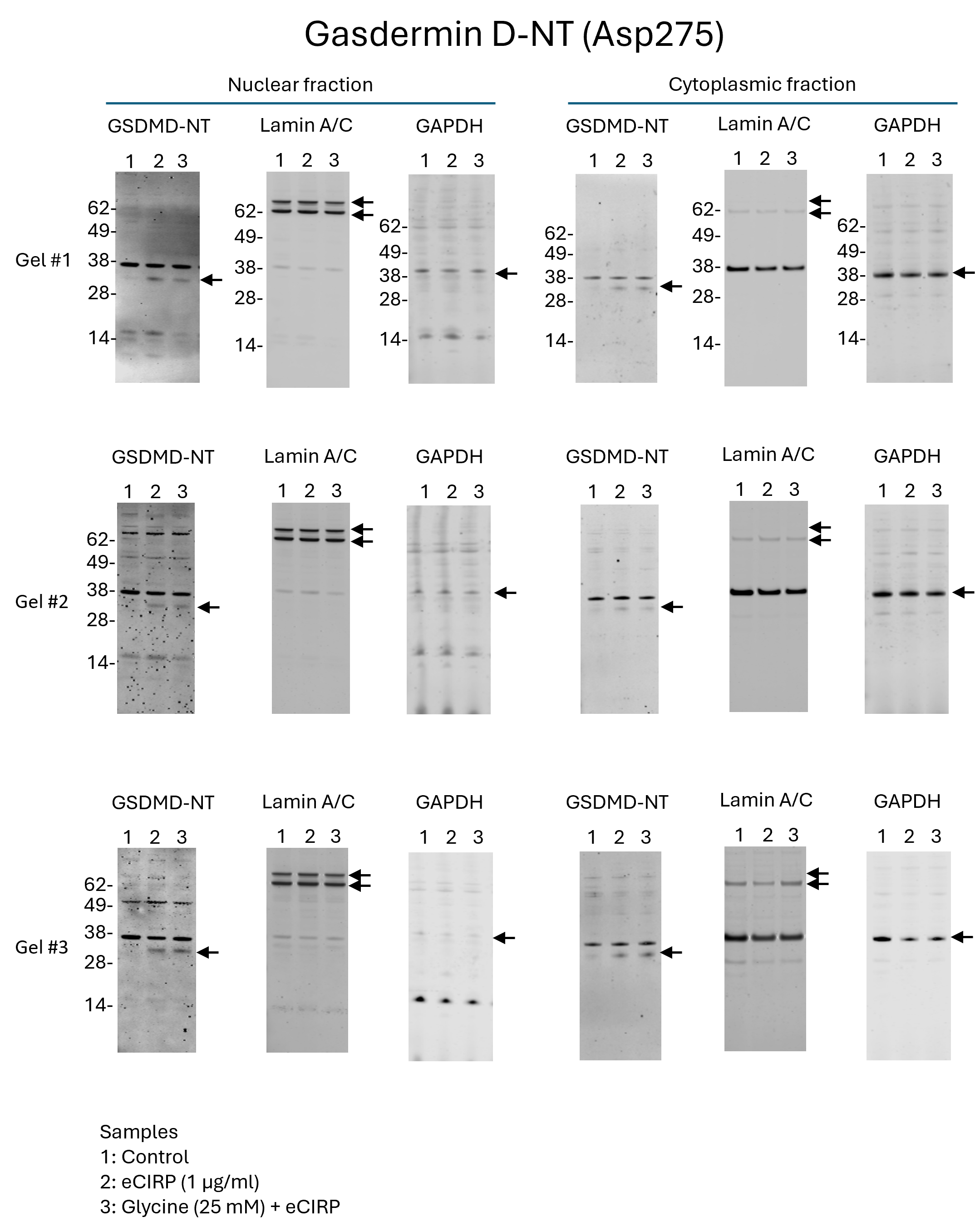

Supplement: Supplementary Figure 4 — western blot analysis of nuclear and cytosolic fraction of the cells. The western blot analysis for the nuclear fractions and cytoplasmic fractions were performed. Each fraction was probed with anti-Asp275 GSDMD-NT, anti-Lamin A/C, and anti-GAPDH antibodies. Each set of data was prepared from three biologically independent experiments. [file Image4.tif]

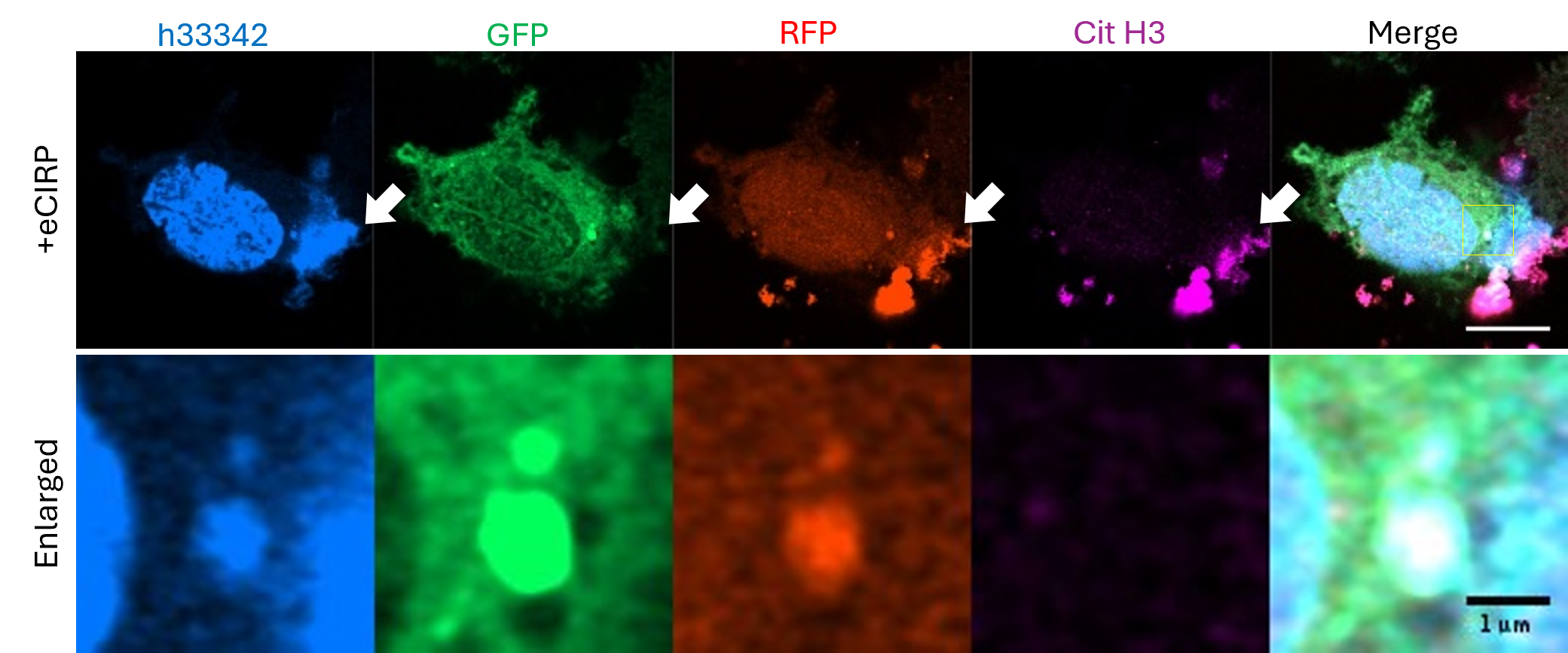

Supplement: Supplementary Figure 5 — Confocal microscopy of RFP::GFP::LC3B and immunofluorescence of Cit H3. The macrophage transduced with RFP::GFP::LC3B was assay by immunofluorescence with anti-Cit Histone3 antibody. METs released from the cell showed intense RFP fluorescence, arrow. The enlarged image showed the cytoplasmic nuclear DNA puncta positive to both of green and red fluorescence. Scale bars are 10 and 1 μm for whole cell and blown up, respectively. [file Image5.tif]

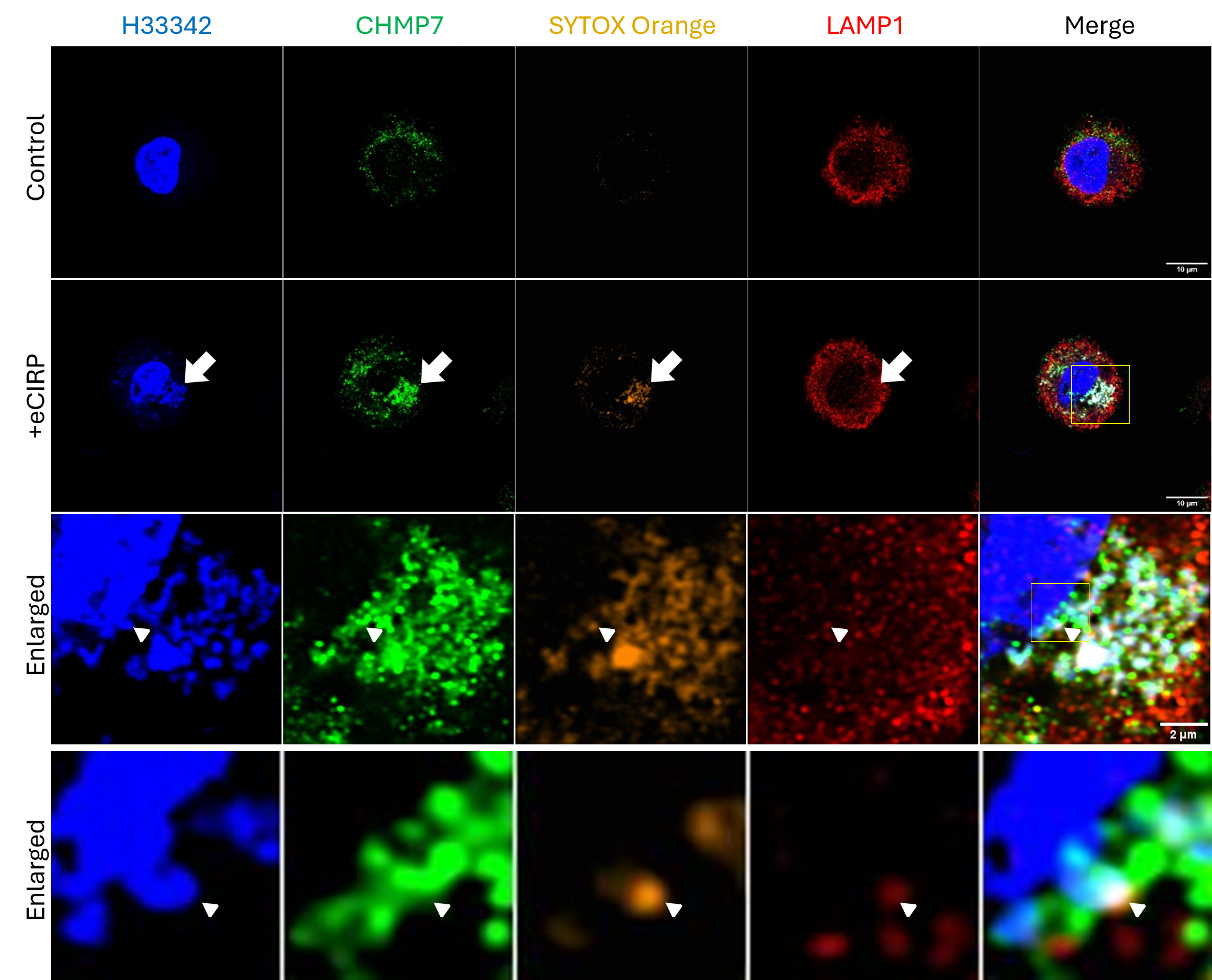

Supplement: Supplementary Figure 6 — DNA release process is accompanied with CHMP7 recruitment. CHMP7 immunofluorescence assay showed that the protein was significantly accumulated in the DNA puncta or proximity to the DNA released, arrow. The blown-up image showed that CHMP7 was recruited to the proximity to the nascent protrusion of nuclear DNA, arrowhead. Notable observation was that the tip of the DNA protrusion had overlapped signals of CMHMP7, SYTOX Orange and LAMP-1 altogether. Scale bars are 10 and 2 μm for whole cell image and enlarged image, respectively. The CHMP7 image with eCIRP treatment was a representative image of 4 different cells. [file Image6.tif]

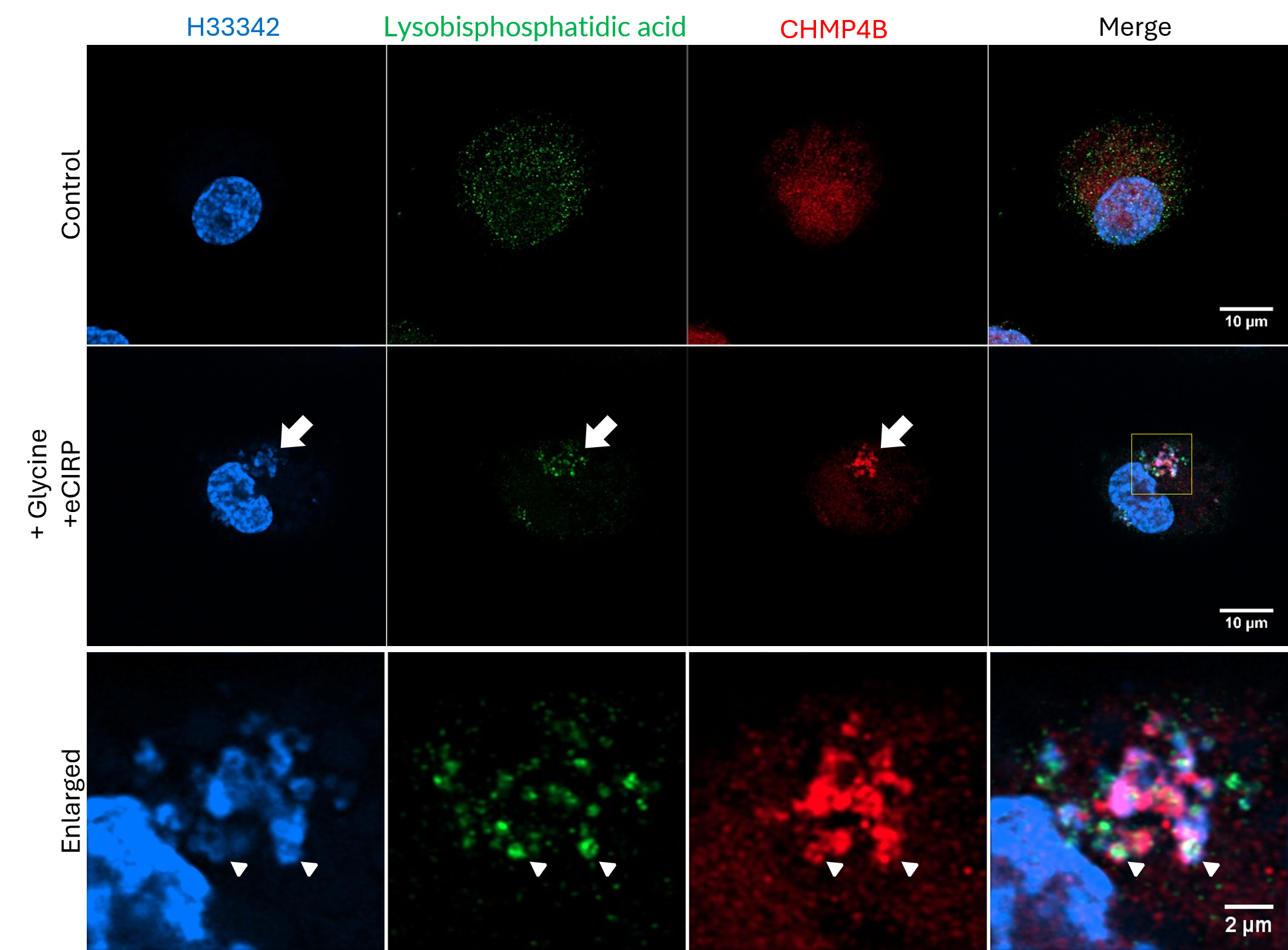

Supplement: Supplementary Figure 7 — Nuclear DNA went through endosomal trafficking. The endosomal marker, lyso-bis-phosphatidic acid (LBPA), was probed by immunofluorescence assay with anti-LBPA antibody. The cell was also stained with anti-CHMP4B antibody simultaneously. Both LBPA and CHMP4B are significantly accumulated where the DNA released from nucleus. The DNA puncta in the enlarged image showed that LBPA signal was in the DNA puncta and CHMP4B decorated the surrounding of the DNA puncta, arrowhead. Scale bars are 10 and 2 μm for whole cell and blown up, respectively. The LBPA image with eCIRP treatment was a representative image of 2 different cells. [file Image7.tif]

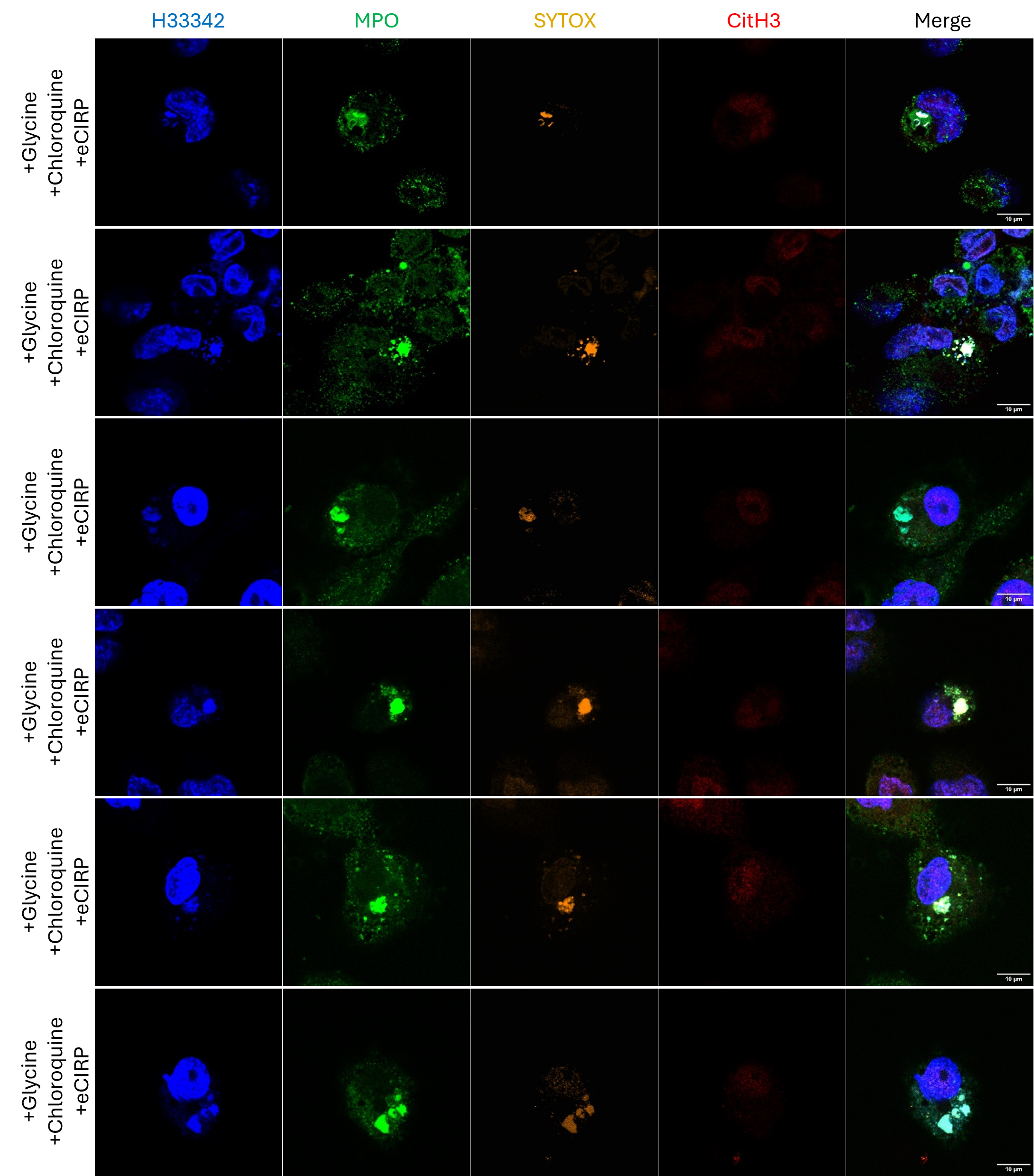

Supplement: Supplementary Figure 8 — Nuclear DNA was significantly accumulated in the cytosol due to the dysfunction of Lysosome by Chloroquine treatment. The treatment of chloroquine (final concentration: 20 μM) to the macrophages was analyzed by immunofluorescence assay using anti-MPO and Cit H3 antibodies. Chloroquine significantly reduced the level of citrullination in the DNA puncta while the MPO is still persistent, arrow. Scale bars are 10 μm. [file Image8.tif]

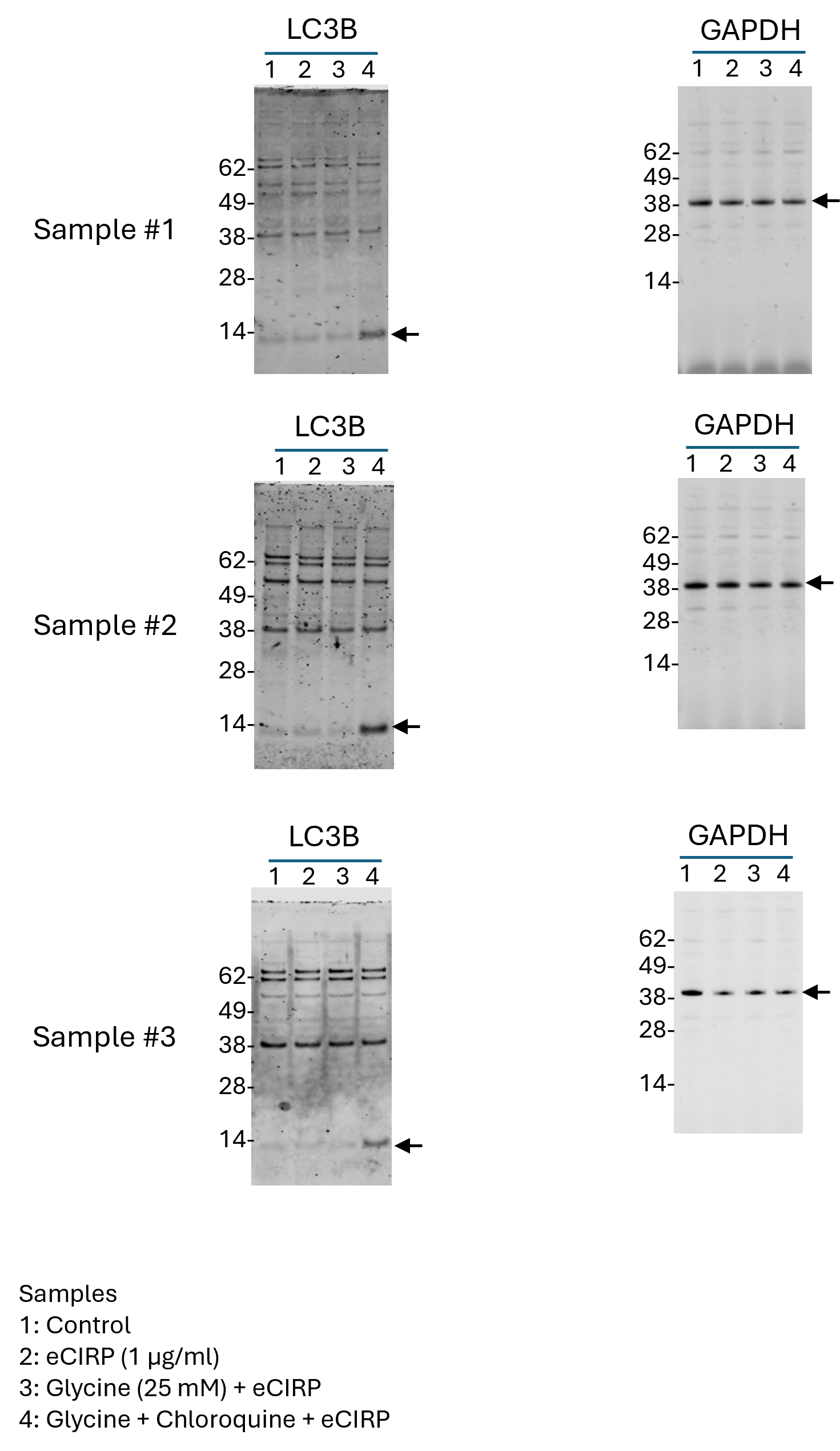

Supplement: Supplementary Figure 9 — LC3B western blot. LC3B level in the cytoplasm was measured by the western blotting with anti-LC3B antibody, which was used for the immunofluorescence assay in Figure 4A . Sample sets were prepared from three biologically independent experiments. [file Image9.tif]

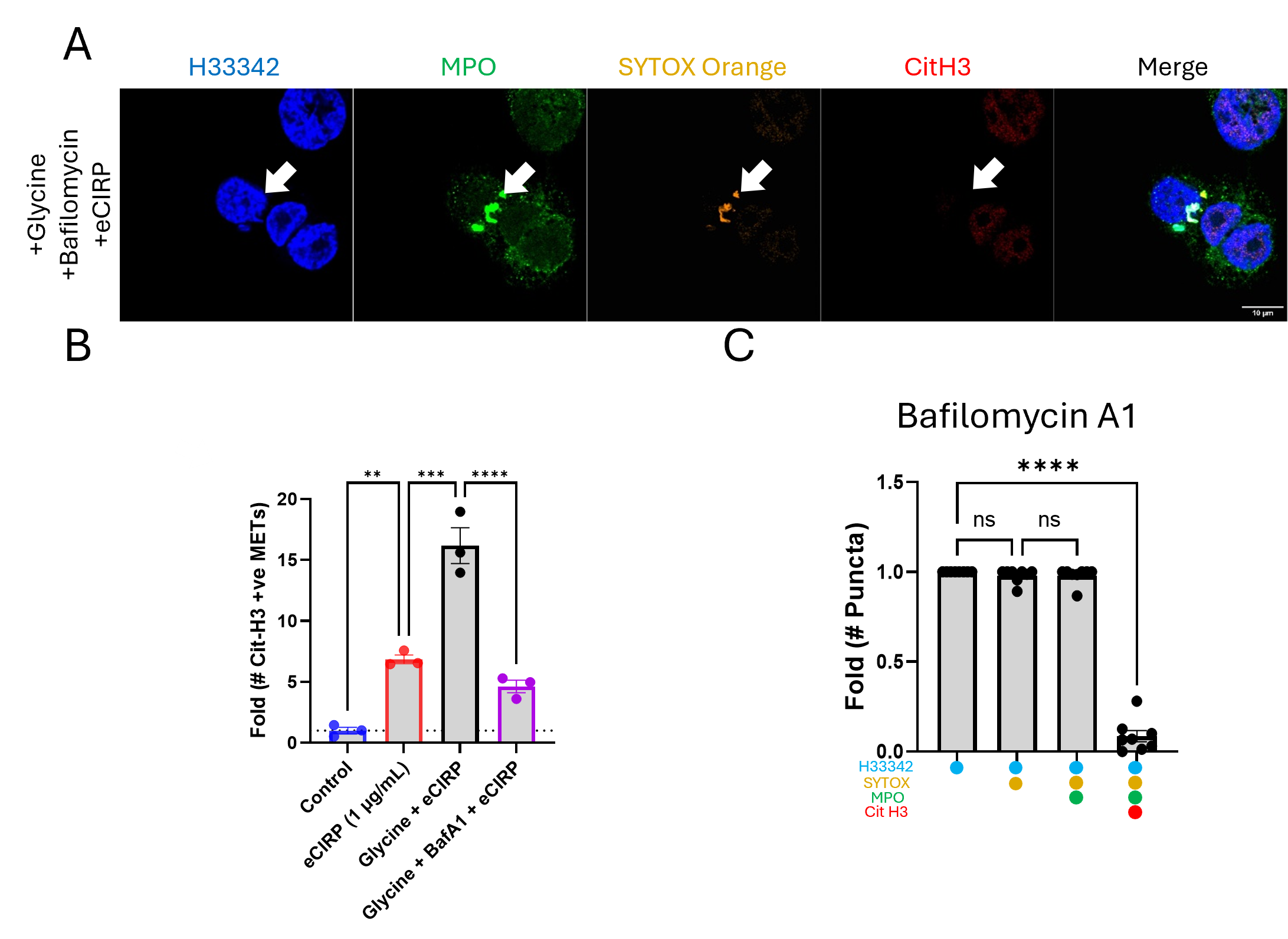

Supplement: Supplementary Figure 10 — Extracellular traps release was also inhibited by bafilomycin A1. The treatment of Bafilomycin A1 (final concentration: 20 nM) to the macrophages was analyzed by immunofluorescence assay using anti-MPO and Cit H3 antibodies, (A). Bafilomycin A1 significantly reduced the level of citrullination in the DNA puncta while the MPO is still persistent, arrow. The quantitative analysis was done with low magnification image analysis with bafilomycin A1 treatment, (B). One-way ANOVA: ** < 0.01 and **** < 0.0001. The analysis of DNA puncta in the cytoplasm of the macrophages treated with bafilomycin A1 showed the lack of citrullination of Histone 3 protein, (C). One-way ANOVA: ns, not significant and **** < 0.0001. [file Image10.tif]

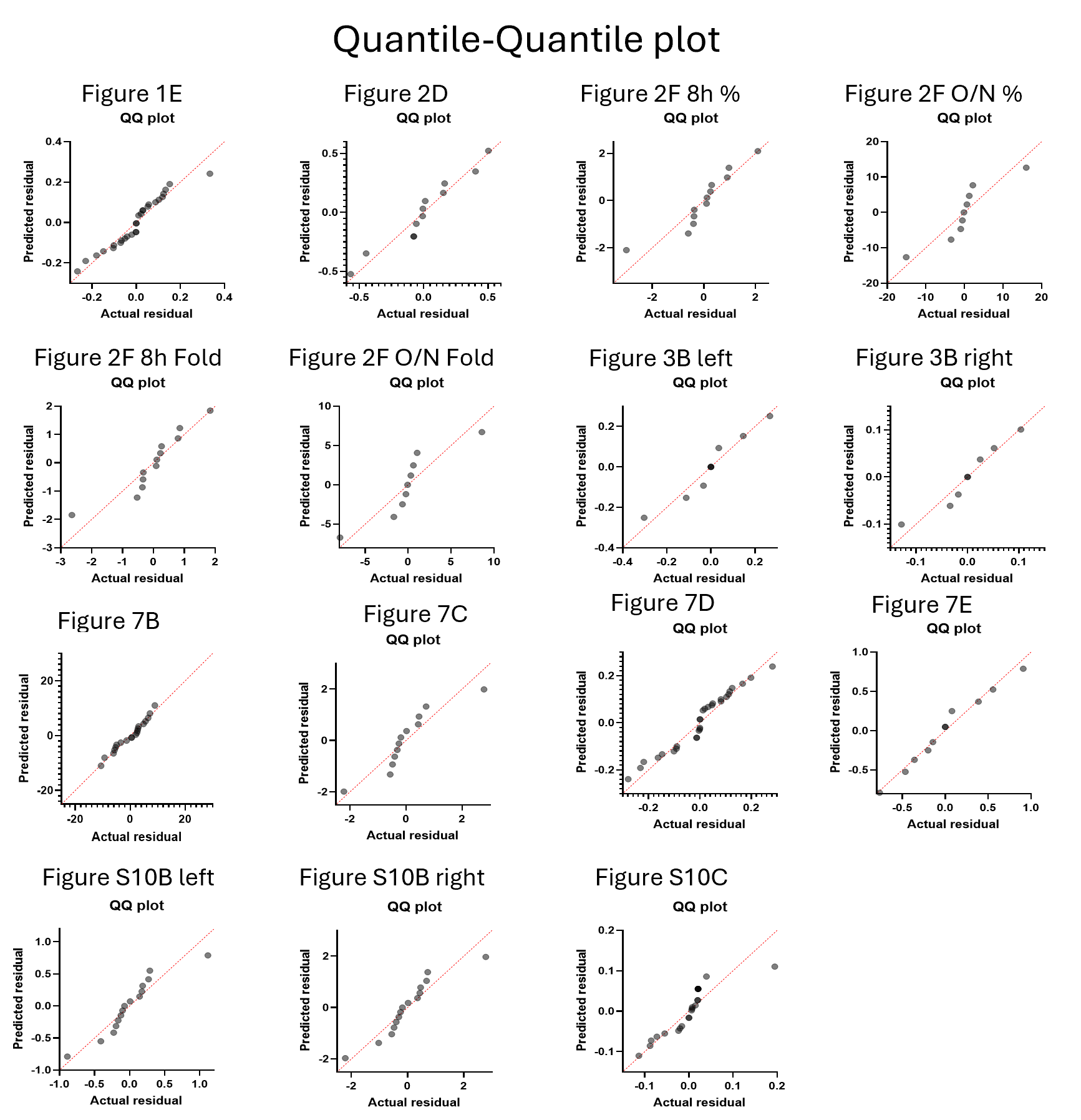

Supplement: Supplementary Figure 11 — Normal distribution test of one way ANOVA analysis. Normal distribution of data for one-way ANOVA analysis was verified by quantile-quantile (QQ) plots and all the analysis showed normal distribution. [file Image11.tif]

Link for movie 1: 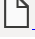 [Movie 1 - 20240724 live imaging.avi](#)

Supplement: Movie S1 — Time-lapse live imaging. The time-lapse microscopy was performed with SYTOX Orange staining. Cells were treated with eCIRP (1 μg/mL) with or without glycine. The time interval of imaging was 15 minutes. The experiment was performed for 16 h. Glycine was treated to the cells 30 minutes prior to the treatment of eCIRP. The time-lapse imaging was started immediately after eCIRP treatment. Red pseudo color was used for the fluorescence signal for SYTOX Orange dye, which dead cell nuclear DNA staining. [file DataSheet1.pdf]

Link for movie 2: 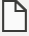 [Movie 2 - Experiment-180-Airyscan Processing-03 -2.mp4](#)

Supplement: Movie S2 — 3 D reconstruction of Confocal z stack image for GSDMD-NT immunofluorescence. The confocal z stack images showed in Figure 3A were used for the 3-D reconstruction. 3-D volume view and animation was generated with Imaris software. Scale bar is 5 μm. [file DataSheet2.pdf]
